# Evolutionary and functional insights into *Leishmania *META1: evidence for lateral gene transfer and a role for META1 in secretion

**DOI:** 10.1186/1471-2148-11-334

**Published:** 2011-11-17

**Authors:** Vidhi Puri, Aneesh Goyal, Rajan Sankaranarayanan, Anton J Enright, Tushar Vaidya

**Affiliations:** 1 Centre for Cellular and Molecular Biology, Council for Scientific and Industrial Research, Uppal Road, Hyderabad - 500 007, Andhra Pradesh, India; 2 EMBL-EBI, Wellcome Trust Genome Campus, Hinxton, Cambridge, CB10 1SD, UK; 3 Syngene International Limited, Biocon Park, Plot No. 2 & 3, Bommasandra Industrial Area-IV Phase, Bommasandra-Jigani Link Road, Bengaluru- 560099, Karnataka, India

## Abstract

**Background:**

*Leishmania *META1 has for long been a candidate molecule for involvement in virulence: META1 transcript and protein are up-regulated in metacyclic *Leishmania*. Yet, how META1 contributes to virulence remains unclear. We sought insights into the possible functions of META1 by studying its evolutionary origins.

**Results:**

Using multiple criteria including sequence similarity, nucleotide composition, phylogenetic analysis and selection pressure on gene sequence, we present evidence that META1 originated in trypanosomatids as a result of a lateral gene transfer of a bacterial heat-inducible protein, HslJ. Furthermore, within the *Leishmania *genome, *META1 *sequence is under negative selection pressure against change/substitution. Using homology modeling of *Leishmania *META1 based on solved NMR structure of HslJ, we show that META1 and HslJ share a similar structural fold. The best hit for other proteins with similar fold is MxiM, a protein involved in the type III secretion system in *Shigella*. The striking structural similarity shared by META1, HslJ and MxiM suggests a possibility of shared functions. Upon structural superposition with MxiM, we have observed a putative hydrophobic cavity in META1. Mutagenesis of select hydrophobic residues in this cavity affects the secretion of the secreted acid phosphatase (SAP), indicating META1's involvement in secretory processes in *Leishmania*.

**Conclusions:**

Overall, this work uses an evolutionary biology approach, 3D-modeling and site-directed mutagenesis to arrive at new insights into functions of *Leishmania *META1.

## Background

Leishmaniasis is a spectrum of parasitic disease caused by *Leishmania spp*. protozoa, transmitted to its mammalian host by bite of an infected female sand fly vector. The clinical manifestations of the disease range from the potentially fatal visceral disease caused by *L. donovani *complex (*L. donovani *and *L. infantum*) to disfiguring, but self curing cutaneous form caused by *L. major *in the Old World and *L. amazonensis *and *L. mexicana *in the New World. Within the sand fly, *Leishmania *exists as an extracellular, flagellated form and gets transformed into an intracellular, sessile amastigote in phagolysosome of the macrophages. The role of many molecules like GP63 [1-5], lipophosphoglycan [6-12], cysteine proteases [13-15] etc. that are differentially expressed in the two developmental stages of the parasite have been studied for their involvement in adaptation of the parasite in such disparate environments and enhancing virulence of the parasite. A lot more remains to be learnt, including the functions, of many such virulence factors. Understanding the mechanistic details of function of virulence factors will help us develop novel intervention strategies against the parasite and the disease.

*META1 *was one such gene identified in *L. major*, found to be conserved across the *Leishmania *genus. Both transcript and protein was found to be up-regulated in the metacyclic stage of promastigotes. The META1 protein was found to localize surrounding the flagellar pocket in *L. major *stationary phase promastigotes [16]. On *META1 *overexpression in *L. amazonensis*, parasites were found to be more virulent than wild-type [17]. Another gene in *Leishmania*, also up-regulated in metacyclic promastigotes is named *META2*, and contains three META domains and a calpain-like domain at the carboxy terminus [18]. META (PF03724) is currently described as a small domain family of unknown function in *Leishmania *META1 and in bacterial proteins, hypothetically secreted/implicated in motility. However, apart from some of its developmental expression and its suggested role in virulence, the precise functions of META1 in the parasite remain unknown.

In order to understand *META1's *function, we investigated its relationship with its near homologs in other organisms. Based on several criteria, we have been able to establish in this study that *META1 *of *Leishmania *is a homolog of a bacterial gene for a heat shock inducible protein, *hslJ *that has been transferred by an ancient lateral gene transfer event between bacteria and a trypanosomatid ancestor. We have highlighted similarities in various characteristics of the two proteins like their relatedness to pathogenicity/virulence, heat inducible expression and also shown by homology modeling that they are not only evolutionarily linked but structurally similar as well. Furthermore, our homology modeling identified yet another structural homolog MxiM, a secretin pilot protein of *Shigella flexneri*, which is involved in that pathogen's type III secretion system. Based on structural homology between META1 & MxiM and subsequent mutagenesis, we have identified a putative hydrophobic cavity in *Leishmania *META1 that seems to be important for function of META1 and indicates a role for META1 in *Leishmania *secretory processes.

## Results

### Sequence similarity searches

The META1 sequence is highly conserved across the *Leishmania *genus [16]. Based on this high degree of conservation and the convenient accessibility of *L. major *genome sequence and other related resources over those for other *Leishmania *species, all of our bioinformatics analyses have used *L. major *META1 as the representative species. We began our study by using BLASTP to search for homologs of META1 protein against the NCBI NRDB. Homologs of META1 were found in all other *Leishmania *species and Trypanosomes (Additional file 1).

**Figure 1:**
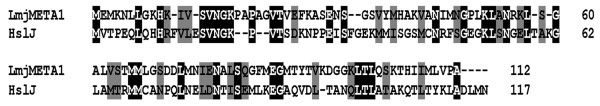
**Sequence similarity between *Leishmania *META1 (1-112 aa) and *E. coli *HslJ (25-140 aa)**. Amino acid sequence of *L. major *META1 (*Lmj*META1) is globally aligned to *E. coli *HslJ devoid of the N-terminal signal sequence using EMBOSS. Residues shaded in black are identical and residues shaded in grey are conservative substitutions. The numbers on the right indicate amino acid positions.

Since BLASTP search failed to detect any functionally characterized homolog within or outside the Trypanosomatidae family, a PSI-BLAST search was performed against NCBI NRDB. This search yielded hits beyond the trypanosomatids as early as the second iteration. Interestingly, these significant hits were all homologs of the heat shock inducible protein HslJ from bacteria. Table 1 lists representative hits after 3 iterations. All sequence homologs of META1 (trypanosomatid as well as bacterial) are characterized by the presence of at least one META domain. Although there is a low direct pairwise sequence identity of 25% between META1 and HslJ (Figure 1 ), bacterial HslJ is currently the closest relative of *Leishmania *META1 outside the family Trypanosomatidae. The most likely explanation for this unusual phyletic relationship is that *META1 *in trypanosomatids originated as a result of a lateral gene transfer (LGT) event from bacteria.

**Table 1:** List of bacterial HslJ hits obtained from PSI-BLAST search after 3 iterations for *Lmj*META1 against non-redundant NCBI database

Species Name	Description	Accession	E-value
*Cyanothece sp. PCC 7822*	Protein of unknown function, DUF306 Meta and HslJ	ZP_03153678.1	7e^-23^

*Beutenbergia cavernae DSM 12333*	Protein of unknown function, DUF306 Meta and HslJ	YP_002881157.1	2e^-16^

*Tolumonas auensis* *DSM 9187*	Protein of unknown function, DUF306 Meta and HslJ	YP_002892522.1	2e^-7^

*gamma proteobacterium HTCC5015*	Conserved hypothetical protein	ZP_05061049.1	2e^-6^

*Roseobacter sp. AzwK-3b*	Hypothetical protein RAZWK3B_01740	ZP_01901204.1	5e^-6^

*Sphingopyxis alaskensis RB2256*	Protein of unknown function, DUF306 Meta and HslJ	YP_617096.1	1e^-5^

*Alcanivorax sp. DG881*	Conserved domain protein	ZP_05041611.1	2e^-5^

*Vibrionales bacterium SWAT-3*	Hypothetical protein VSWAT3_19198	XP_844891.1	2e^-5^

*Roseovarius sp. TM1035*	Probable secreted protein containing HslJ-like protein	XP_844900.1	2e^-5^

*Colwellia psychrerythraea 34H*	Putative lipoprotein	XP_001464756.1	3e^-5^

*Beijerinckia indica subsp. indica ATCC 9039*	Protein of unknown function, DUF306 Meta and HslJ	YP_001832384.1	3e^-5^

*Bacteroides vulgatus* *ATCC 8482*	Hypothetical protein BVU_2381	YP_001299662.1	5e^-5^

*Hyphomonas neptunium ATCC 15444*	hypothetical protein HNE_0979	YP_759703.1	6e^-5^

*Opitutaceae bacterium TAV2*	Heat shock protein-like protein	ZP_03723590.1	9e^-5^

### Anomalous nucleotide composition

**Figure 2:**
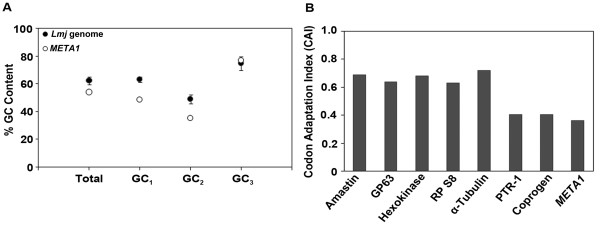
**Atypical nucleotide composition**. **(A)** GC content anomaly. GC content anomaly. Percentage of total GC content and GC content at codon positions of all *L. major *ORFs and *L. major META1*. The vertical error bars represent the mean value +/- 2 S.D. for *L. major *genome. Total, total GC content; GC_1_, GC content at first codon position; GC_2_, GC content at second codon position and GC_3_, GC content at third codon position. **(B)** Codon usage bias. Codon Adaptation Index (CAI) of 7 reference genes (Amastin, GP63, Hexokinase, Ribosomal Protein (RP) S8, α-Tubulin, PTR-1, Coproporphyrinogen (Coprogen)) and META1. The accession numbers of the protein sequences used are given in Additional file 2.

Most genomes have distinct overall GC content and the genes in their genome are fairly similar in base composition and patterns of codon usage. A laterally transferred gene continues to carry imprints of its origin and ancestry whilst residing in its current recipient genome. Such 'foreign genes' can be identified by their unusual sequence characteristics vis a vis the recipient genome [19-21]. GC content at first and third codon positions and synonymous codon usage are generally used for such analysis. ORFs are identified as atypical if their GC contents at first and third codon positions (GC_1 _& GC_3 _respectively) are two or more standard deviations (SDs) higher or lower than the respective means for all genes in that genome. Our analysis revealed that the total GC content of *L. major META1 *is 54%, which is 6.3 SD lower than the average of the total GC content of all genes in *L. major *genome. In addition, *LmjMETA1 *has a GC_1 _content of 48.67% (15.8 SD lower than GC_1 _of all genes in *L. major *genome) and a GC_3 _content of 77% (within the range of SD for GC_3 _of *L. major *genome), as summarized in Figure 2A. GC content anomaly results establish that *META1 *nucleotide composition is distinct from the overall *Leishmania *genome and therefore, is possibly of foreign origin.

Codon adaptation index (CAI), on the other hand, gives an account of similarity of synonymous codon usage to that of a standard set of highly expressed genes for that organism. Thus, for a native gene CAI is high and for a gene of exogenous origin CAI value is low. As part of CAI determination, we analyzed a set of other genes for reference along with *META1* (Additional file 2 ). The genes that were included: Amastin & GP63 (specific to trypanosomatids), Hexokinase & Ribosomal Protein S8 (present in all taxa), α-Tubulin (a pan-eukaryotic structural protein) and PTR-1 & Coproporphyrinogen, previously predicted to be laterally transferred in *L. major *from bacteria [22]. *META1*, PTR-1 and Coproporphyrinogen all have low CAI values when compared to the other reference genes (Figure 2B ), suggesting again, that *META1 *is of exogenous origin. We also carried out a similar analysis on META1 homologs in *T. cruzi* (Additional file 3 ); where once again the CAI of the META homologs is lower than that of other *Trypanosoma *genes (RP S8 and α-Tubulin). These analyses, and the striking frequency of bacterial HslJ hits, point to bacterial *hslJ *being the exogenous source of *META1 *into trypanosomatids.

### Phylogenetic relationship between META1 and HslJ

**Figure 3:**
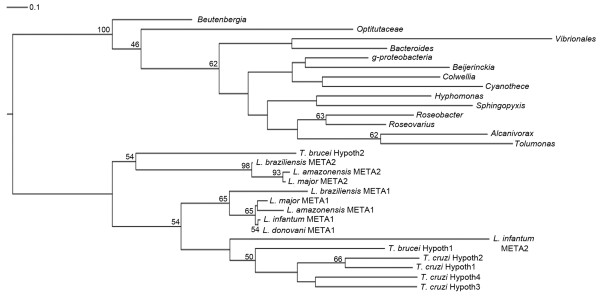
**Phylogenetic relationship between META1 and HslJ**. Phylogenetic analysis was done using PhyML [68]. The phylogram represented is a consensus of a 100 bootstrap replicates and is rooted at the basal node. The numbers at the node represent the percentage of trees with the same node among all of the bootstraps. Only bootstrap values ≥ 40% have been mentioned. A horizontal bar marked with 0.1 above the tree represents scale of the generated tree.

In order to study the phylogenetic linkage between META1 and HslJ, we generated a phylogenetic tree based on the protein sequences of META1 homologs in trypanosomatids and HslJ homologs in bacteria. A multiple sequence alignment containing 30 sequences (16 trypanosomatid and 14 bacterial) was used to create a phylogenetic tree using PhyML. Topology of the phylogenetic tree and the bootstrap values of the branch points (Figure 3 ), again indicate the occurrence of a LGT event of *META/hslJ *from bacteria to a trypanosomatid.

### Synonymous and non-synonymous rate estimates

**Figure 4:**
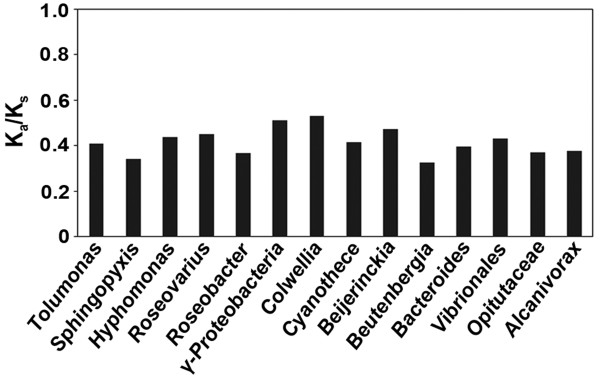
**Synonymous (K_s_) and non-synonymous (K_a_) substitution rates of *Lmj*META1 and META domain of all bacterial HslJs**. Substitution rates were estimated using Yang and Nielson method [23] as implemented in yn00 in the PAML package. All bacterial HslJ homologs from PSI-BLAST with a representative sequence from each genus were used, with an e-value threshold ≤ 1x10^-5^.

Estimation of synonymous (K_s_) and non-synonymous substitution (K_a_) rates is an important tool for understanding molecular sequence evolution and the ratio of non-synonymous to synonymous rate of substitution (K_a_/K_s_) reflects the selection pressure on a gene [23]. We observed that *META1 *and the META domain in bacterial *hslJ *homologs are under strong purifying selection as indicated by the K_a_/K_s _values < 1 in a pairwise comparison of *LmjMETA1 *and META domain of 14 bacterial *hslJ *homologs (Figure 4). Therefore, lower substitution rates for *META1 *emphasizes the selection pressure on it, a constraint on the *Leishmania *genome not to change it rapidly and thus, the importance of conservation of this gene.

### META1 expression in *Leishmania*

**Figure 5:**
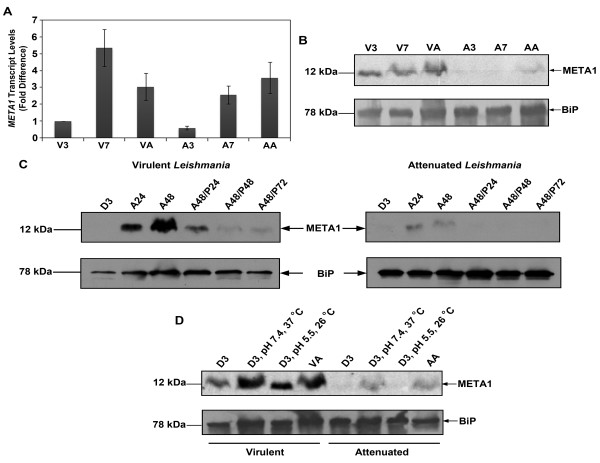
**META1 expression in *Leishmania donovani***. **(A)** Quantitative RT-PCR analysis of *META1 *transcript in virulent and attenuated lines. The relative expression of *META1 *gene in various stages is presented as fold difference over expression in V3. The average of 3 biological replicates is presented. Bar represents standard deviation. V3 and A3: virulent and attenuated log phase promastigotes respectively; V7 and A7: virulent and attenuated stationary phase promastigotes respectively; VA and AA: virulent and attenuated amastigotes respectively. **(B)** META1 protein expression in virulent and attenuated lines. Total cell lysates from various stages of *L. donovani *were obtained at the indicated growth stages. The samples were assessed by western blotting with META1 (upper panel) and BiP (lower panel) antibodies. *Leishmania *BiP is a chaperone protein in the endoplasmic reticulum and was used as a loading control. **(C)** Time-course kinetics of META1 expression in promastigotes and amastigotes. Total cell lysates at various time intervals after switch to amastigote culture conditions from promastigotes of *L. donovani *were obtained at the indicated time points. Lysates were probed with META1 (upper panel) and BiP (lower panel) antibodies. Left panel represents virulent and right panel represents attenuated *L. donovani *respectively. D3: log phase promastigotes; A24 and A48: 24 hours and 48 hours after shift to amastigote culture conditions respectively; A48/P24, A48/P48 and A48/P72: 48 hours amastigotes shifted to promastigote conditions for 24 hours, 48 hours and 72 hours respectively. **(D)** Heat inducible expression of META1. Total cell lysates after individual or concomitant shift to pH 5.5 or 37°C for 48 hours of *L. donovani *promastigotes at pH 7.4 and 26°C were obtained for both virulent (lanes 1-4) and attenuated (lanes 5-8) *L. donovani*. Lysates were probed with META1 (upper panel) and BiP (lower panel) antibodies. D3, pH7.4, 26°C: log phase promastigotes; D3, pH7.4, 37°C and D3, pH5.5, 26°C: log phase promastigotes at pH 7.4 shifted to either 37°C or pH 5.5 at 26°C for 48 hours respectively; VA and AA: virulent and attenuated amastigotes respectively.

*hslJ *transcript is up-regulated in more pathogenic strains of *E. coli *[24] and the protein is heat inducible [25]. To delineate similarities between *META1 *and *hslJ*, we investigated the expression pattern of *META1. META1 *transcript levels were determined by QRT-PCR at different developmental stages of virulent and attenuated lines of *L. donovani. META1 *transcript is up-regulated from log to stationary phase promastigotes as well as from promastigote to amastigote stage (Figure 5A ). META1 protein in *L. donovani *is maximally expressed in amastigotes (Figure 5B**) **even though *META1 *transcript levels in amastigotes are lower than stationary phase promastigotes. META1 protein is induced within 24 hours of shift to amastigote culture conditions, increases further upto 48 hours and its expression decreases within 24 hours of reversal back to promastigote conditions (Figure 5C ). The most striking observation is that attenuated *Leishmania *have reduced *META1 *expression under all conditions, as compared to virulent cells (Figure 5).

Acidic pH and increased temperature (shift from 26°C to 37°C) are very important cues for the *in vivo *as well as *in vitro *conversion of promastigotes to amastigotes [26-28]. Since we found that META1 is up-regulated in amastigotes stage, we investigated the role of these two parameters individually. We found that while both acidic pH and increased temperature induce META1 expression, increased temperature has a greater effect (Figure 5D ). Levels of META1 on western blots have been quantified as shown in Additional file 4.

### Structural homology between META1 and HslJ

**Figure 6:**
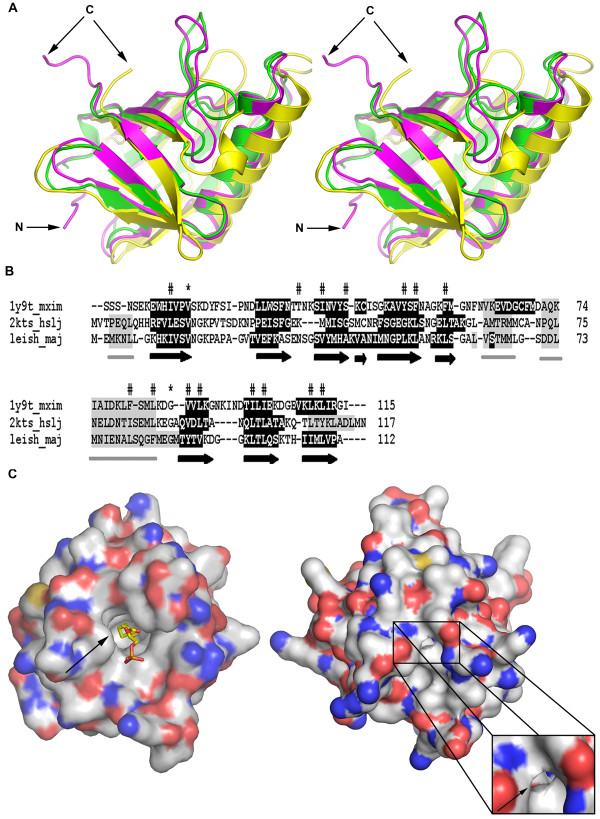
**Structural similarities between META1, HslJ and MxiM**. (A) Superposition of META1 (green), HslJ (magenta; PDB code 2kts) and MxiM (yellow; PDB code 1y9t) in stereoscopic view. The arrowheads mark the N-terminus and the C-terminus of the proteins. The figure was prepared using the software PyMol [73]. (B) META1 and HslJ sequence alignment based on MxiM structural superposition. Regions marked by horizontal grey bars below the alignment indicate helices and its corresponding amino acids are also shaded in grey. Beta strands are marked by black bold arrows below the alignment and the corresponding amino acid residues are shaded in black. Conserved residues are marked by (*) above the alignment. Amino acid residues lining the core of the hydrophobic cavity deduced based on structural superposition of META1 and HslJ with already described pocket of MxiM, are marked by (#). The alignment was generated by PROMALS3D [63]. (C) Molecular surface representation of MxiM (1y9t, left panel) and *Leishmania *META1 (right panel) highlighting their respective hydrophobic cavities. This representation was generated by superposing *Leishmania *META1 (right panel) onto MxiM (left panel, 1y9t) with its ligand in the proposed hydrophobic cavity. The surface is colored according to atom type: carbon atoms in grey, sulphur atoms in yellow, nitrogen atoms in blue and oxygen atoms in red. A zoom in view of the putative hydrophobic cavity of *Leishmania *META1 is represented as inset. Hydrophobic cavities of the both proteins have been indicated by an arrow. First 4 amino acids from the N-terminus from *Leishmania *META1 have been removed for clarity. The figure was prepared using the software PyMol [73].

In the absence of a solved structure of *Leishmania *META1 protein, we used 3D-JURY [29] structure prediction to look for structural homologs of META1. Consistent with our earlier searches, we found the top scoring model for META1 was based on *E. coli *HslJ. HslJ structure has 10 β-strands, which resembles a β-barrel structure except of the discontinuity caused due to the presence of the α-helix. We also generated a structural model by homology modeling using MODELLER 9v2 [30] for *Leishmania *META1 using *E. coli *HslJ (PDB code 2kts) as the template. The final model (Figure 6A) comprises of complete META1 sequence (1-112) threaded on known NMR structure of *E. coli *HslJ (PDB code 2kts) for residues 25-140 (Figure 6B). The two proteins share a similar structural fold given that 107 C_α _atoms of total 116 C_α _atoms across the length of the proteins could be superposed with a root mean square deviation (RMSD) value of 1.2 Å and a sequence identity of ~ 28%. The striking structural similarity underscores that despite low sequence identity, META1 and HslJ have been under evolutionary constraints to retain a similar fold.

### Comparison to proteins with similar folds of known function

Since little is known about *E.coli *HslJ's function with respect to its structure, we decided to look for additional proteins with similar fold. In DALI server [31], we found a structural homolog of HslJ: the *Shigella flexneri *protein MxiM (Figure 6A and Additional file 5 ). Despite a low sequence identity between HslJ and MxiM of only ~13%, 94 C_α _atoms of the total 116 C_α _atoms could be superposed with an RMSD value of 2.5 Å, as determined by DALI pairwise comparison [32]. Similarly, META1 and MxiM share a sequence identity of ~9%; yet, 94 C_α _atoms out of the total 115 C_α _atoms could be superposed with an RMSD of 2.6 Å. MxiM is a type III secretion apparatus (TTSS) pilot protein, which is a 142-residue lipoprotein, essential for the assembly and membrane association of the *Shigella *secretin, MxiD [33]. A deficiency in MxiM leads to complete loss of TTSS function and virulence [34]. The structure of MxiM contains a 'cracked barrel' motif that creates a striking cleft in the centre of the protein. The cavity is entirely hydrophobic in nature and binds to lipid moieties of outer membrane (OM) of the bacteria [35]. Likewise, we observed that META1 and HslJ also have a similar hydrophobic cavity upon their superposition to MxiM (Figure 6C ). However, unlike in MxiM, the putative hydrophobic cavity in META1 has at least one charged residue in its core (Figure 6B ). Overall, structural comparisons between META1, HslJ and MxiM further reinforce their fold similarity. This pattern of structural and not sequence conservation signifies selection pressure on the structure of these proteins, probably for maintaining similar functions.

### META1 is involved in secretory processes in *Leishmania*

**Figure 7:**
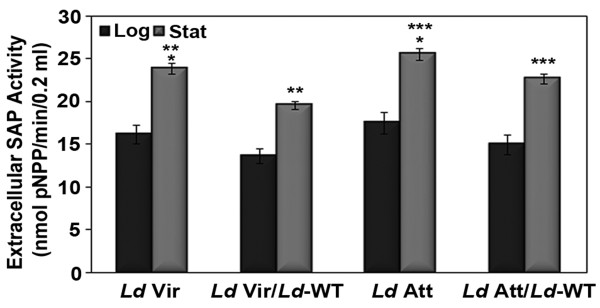
**META1 is involved in secretion in *Leishmania***. Extracellular SAP activity at log (black bars) and stationary phase (grey bars) of wild-type (*Ld *Vir &* Ld *Att) and META1 overexpressing (*Ld *Vir/*Ld-*WT &*Ld *Att/*Ld-*WT) *L. donovani *virulent & attenuated promastigotes lines respectively. SAP activity is represented in nmoles of pNPP hydrolyzed per min per 0.2 ml. The average of at least 5 biological replicates is presented. Bar represents the standard deviation. Means of the respective values at stationary phase were compared for statistical significance using a paired Student's t-test. *P < 0.005, **P ≤ 0.0002 and ***P ≤ 0.005.

In *L. major*, META1 is reported to localize around the flagellar pocket [16], the site for exo- and endocytosis in *Leishmania *[36]. MxiM, in addition to its known participation in *Shigella *TTSS, is also known to be structurally similar to the bacterial lipocalin [35]. Lipocalins are known extracellular transporters that bind small hydrophobic molecules [37]. Against this background of information, the striking structural similarity between META1 and MxiM prompted us to examine whether *Leishmania *META1 had a role in *Leishmania *secretory processes. Secreted acid phosphatase (SAP) is the most abundant secreted glycoprotein of *L. donovani *[38-40] and estimation of extracellular SAP activity is routinely used as an indicator of the status of secretory processes in the parasite [41-43]. Therefore, SAP activity was assayed in culture supernatants in the context of different levels of META1. Extracellular SAP activity was found to be lower at both log and stationary phase in *L. donovani *virulent promastigotes that have more META1 protein, when compared to attenuated promastigotes (Figure 7). In order to determine whether extracellular SAP activity is directly related with levels of META1 expression, we ectopically expressed *META1 *in both virulent and attenuated *L. donovani *promastigotes. The ectopic copy of *L. donovani META1 *protein was tagged with GFP at the C-terminus to distinguish from the endogenous protein. On META1 overexpression, extracellular SAP activity further reduced in both virulent and attenuated overexpression lines in comparison to their respective wild-types (Figure 7 ). Overexpression of META1 at protein level and its effect on growth kinetics is shown in Additional file 6. This suggests that cells having higher amounts of META1 protein have lower extracellular SAP activity, indicating a role for META1 in secretory processes in *Leishmania*.

### Mutations in putative hydrophobic cavity of META1 alter secretion in *Leishmania*

To examine whether the predicted hydrophobic cavity has any role in META1's function, we decided to mutate smaller hydrophobic residues in the cavity to bulkier ones, wherein we replaced leucines at 58^th ^and 80^th ^position with a phenylalanine individually and both simultaneously (L58F or/and L80F). L58 is located at the entry of the cavity and is conserved across trypanosomatids and bacterial HslJ. On the other hand, L80 is inside the hydrophobic cavity and is conserved in trypanosomatids, with the exception of *L. braziliensis *and *T. cruzi *as shown in Additional file 7. Mutant proteins were C-terminally tagged with GFP, in order to distinguish from endogenous protein and were ectopically expressed in *L. donovani *promastigotes.

**Figure 8:**
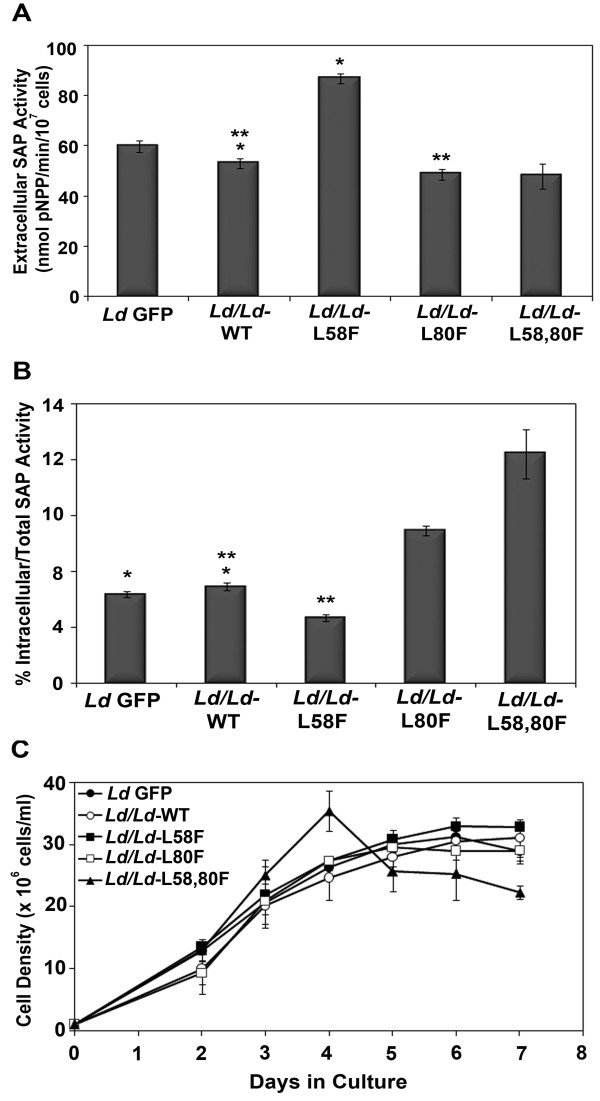
**Mutagenesis in putative hydrophobic cavity of META1 alters secretion in *Leishmania***. (A) Mutations in putative hydrophobic cavity of META1 alter extracellular SAP activity. Extracellular SAP activity in *L. donovani *at stationary phase of vector control (*Ld *GFP), wild-type META1 overexpression line (*Ld*/*Ld-*WT), mutant META1 overexpression lines: L58F (*Ld*/*Ld-*L58F); L80F (*Ld*/*Ld-*L80F) and L58,50F (*Ld*/*Ld-*L58,80F) respectively. SAP activity is represented as nmol/min/10^7 ^cells. Means of the respective values were compared for statistical significance using a paired Student's t-test. *P < 0.0001 and ** P < 0.0003. (B) Fraction of intracellular v/s total SAP activity. Results are expressed as percentage of intracellular SAP activity of total SAP activity (intracellular and extracellular) measured in nmol/min/10^7 ^cells in all the transfectants at stationary phase. Means of the respective values were compared for statistical significance using a paired Student's t-test. *P < 0.004 and ** P < 0.0001. (C) Growth kinetics of *L. donovani *on META1 overexpression. Growth curves of *L. donovani *overexpressing either the wild-type META1, *Ld/Ld*-WT or mutant META1 L58F, L80F and L58,80F were compared to that of *Leishmania *with control vector, *Ld *GFP. Cell density (x 10^6 ^cells/ml) of each culture was determined at 24 hour time intervals after 48 hours of initial inoculation for up to 7 days. The cell densities plotted are average of at least 3 biological replicates.

Extracellular SAP activity increased by 40% in *L. donovani *overexpressing L58F mutant META1 protein, as compared to cells overexpressing the wild-type (WT) META1 (Figure 8A ). However, L80F mutation in META1 decreased the extracellular SAP activity with respect to WT overexpression. Moreover, extracellular SAP activity of the double mutant L58,80F was found to be similar to that of L80F mutant in *L. donovani* (Figure 8A ). We also determined the intracellular SAP activity in all of these *Leishmania *lines. Consistent with the changes in extracellular activity, the percentage of intracellular v/s total SAP activity is higher in cells overexpressing WT, L80F and L58,80F META1, but lower in the L58F mutant, as compared to GFP control (Figure 8B ). These results suggest that the 58^th ^and 80^th ^position are critical for the function of META1 in *L. donovani*. Similar results were obtained when L58F and L80F mutations were introduced into *L. major *META1, either individually or simultaneously (Additional file 8 ), reiterating that the *L. donovani *and *L. major *META1 proteins have a conserved function. We also observed a growth defect only in the double mutant in both *L. donovani *and *L. major*, which have higher multiplication rate at late logarithmic phase and start declining sooner than WT overexpression cells (Figure 8C and Additional file 8 respectively). The difference in SAP activity between cells overexpressing WT versus META1 mutant forms was not due to different amounts of META1 protein being expressed, as shown by western blots (Additional files 6 and 8 ). In order to eliminate the possibility of misreporting extracellular SAP activity due to cell lysis, a western blot on culture supernatants was done with GP63 and BiP antibodies, along with whole cell lysates as control. The culture supernatants that we used for the experiment did have extracellular GP63 but lacked ER chaperone BiP, as shown in Additional file 9. Overall, alteration in extracellular SAP activity in cells expressing META1 mutants points to a role for META1 in *Leishmania *secretory processes and highlights the importance of the putative hydrophobic pocket to META1's function.

## Discussion

Despite *Leishmania *META1's well known association with virulence, its functions have remained poorly understood. We have attempted to elucidate the functions of META1 by identifying and examining its known homologs in other organisms. We have observed an unusual phyletic relationship between bacterial HslJ and trypanosomatid META1 which highlights LGT as the most rational explanation for the origin of *META1 *sequences in trypanosomatids.

Analyses of several pathogen genomes suggest a widespread occurrence of LGT [20,22,44-49]. These studies have relied on combinations of aberrant nucleotide composition or sequence similarity searches and phylogenetic analyses. In our study we have included all of these approaches: sequence similarity, overall GC content bias, GC content bias at 1^st ^and 3^rd ^codon positions and codon usage bias of *META1 *over whole genome of *L. major *and phylogenetic distribution pattern. All of the individual analyses are consistent with the notion of *META1 *being laterally transferred from bacteria into trypanosomatids (Figures 2 and 3 ). In the analyses of such LGT events it is crucial to factor in multiple lines of evidence [50]. By itself, the phylogenetic tree may be open to additional interpretations such as a "reverse" LGT flow from trypanosomatids into bacteria or a retention of META1 in trypanosomatids, from a common ancestor (with bacteria) with a loss from other species. However, the consistent topology of the phylogenetic tree, rooted in multiple manners (Figure 3 and Additional file 10**) **taken together with the striking anomalous nucleotide composition of META1 in *Leishmania *make it unlikely that the LGT flow of META1 was from trypanosomatids into bacteria. Or, that META1 was retained within *Leishmania *from a common ancestor: if META was persisting from an ancient common ancestor, its GC composition would have been assimilated into the trypanosomatid genomes. However, we cannot exclude a possibility that META1 was present in an ancient common ancestor, was lost during the evolutionary emergence of eukaryotes and at some point, regained by trypanosomatids through a LGT from bacteria. Clearly, more definitive answers to such events will emerge as the genome sequence information coverage of various taxa expands.

Analyses of anomalous nucleotide composition is quite effective in the detection of recent LGT events but becomes handicapped as the time scale of the event in question increases typically because the gene in question has more time to adjust to the host genome [50]. Given the diversity of bacterial species that have the META domain and the limited occurrence of META only in trypanosomatid eukaryotes, it is most likely that the transfer of META1 into trypanosomatids was an ancient event. In this regard, it is striking that the anomalous nucleotide composition of *Leishmania *META1 continues to be easily discernible.

Maintenance of the foreignness of a laterally transferred gene in the context of its recipient genome could reflect either a recent transfer event or a selective pressure to maintain certain features. Given the occurrence and conservation of *META1 *sequence throughout trypansomatids, it is most likely the transfer of META1 from bacteria was an early event into a trypanosomatid ancestor. An organism always maintains certain evolutionary constraints on its essential genes, in order to prevent loss of function caused due to mutations. Thus, a gene under purifying selection will evolve at a slower rate than a gene under positive selection. Upon comparison of *META1 *with its bacterial homologs, we found that *META1 *is under strong purifying selection, therefore, highlighting the importance of function of this gene for the organism (Figure 4).

Selection constraints on the META1/HslJ pair also underline the possibility of having similar properties. Our results have highlighted some of the properties shared by HslJ and META1. *hslJ *transcript up-regulation was found to be associated with pathogenicity in *E. coli *[24] and recovery of heat injured *Salmonella enteritidis *[51]. In comparison, we observed that *META1 *expression too is regulated in developmental stages in *L. donovani*, consistent with earlier reports [17]. We have also shown that attenuated *Leishmania *have lower META1 expression than virulent cells, consistent with a role for META1 in *Leishmania *virulence. In *Leishmania*, META1 expression is inducible by both temperature and acidification, with the temperature-mediated change being more significant (Figure 5 ). This observation underscores the heat-inducible property of META1, like its bacterial homolog HslJ [25].

HslJ has been associated with increased resistance against a gyrase inhibitor novobiocin [52]. We examined in *Leishmania *if META1 expression is associated with novobiocin resistance in virulent & attenuated lines of *Leishmania *as well as in META1 overexpressing lines. In the above mentioned conditions, we found no such correlation (data not shown). Clearly, not all functions are common between META1 and HslJ. Recently, *L. amazonensis *META2 that has 3 META domains and a C-terminal calpain-like domain was reported to be implicated in novobiocin resistance [53]. This raises the possibility that our observations may be explained by META2 complementing for novobiocin resistance independent of META1 expression levels.

The 3D-modeling studies of META1 allowed us to identify an additional structural homolog of META1, the secretin pilot protein of *Shigella flexneri*, MxiM (Figure 6A ). These three proteins: META1, HslJ and MxiM share a strikingly similar fold, in spite of a very low sequence identity. The essential features about the structural similarity of these proteins highlighted upon superposition are: a common cracked barrel motif and a set of conserved hydrophobic residues in the central pocket of the protein (Figure 6B ). Such a structural homology between META1, HslJ and MxiM suggests a possibility of similar function of these proteins. Many proteins with similar function have been known to retain similar structural folds despite very low sequence identity and ambiguous secondary structure prediction [54,55]. The hydrophobic cavity in MxiM is known to bind lipid moieties of bacterial membranes. However, unlike MxiM, the putative cavity of META1 has at least one charged amino acid suggesting that the ligand of this putative pocket may be different from MxiM (Figure 6B). It is possible that these proteins participate in similar activities, with certain residues in the cavity defining specificity of the ligand.

Changes in levels and predicted structure of META1 affect the quantum of extracellular activity of the well characterized marker for *Leishmania *secretory processes, SAP (Figures 7 and 8 ). A comparison of proportion of intracellular v/s total SAP activity shows that overexpression of either WT or mutant META1 changes the extracellular SAP secretion (Figure 8B ). However, while ectopic expression of L80F mutant and WT reduces extracellular SAP activity, the L58F mutant META1 caused an increase in extracellular SAP activity. The L58F mutation appears to act as a loss of function event: there is more META1 protein but with a reduced negative effect on SAP activity. The proximity of this 58^th ^position to the entrance of the putative hydrophobic cavity (Additional file 7**) **suggests impairment in interactions with the probable ligand of the putative cavity that may interfere with META1's function, in turn interfering with META1's ability to suppress SAP secretion. Additionally, in *L. donovani*, L58 epitopic region seems to be highly antigenic: when lysates of cells overexpressing the different forms of META1 were probed with polyclonal META1 antisera on a western blot, the L58F reactivity was extremely low compared to WT overexpression control. However, when the same proteins, all of which have GFP tagged to their C-termini, were probed with polyclonal GFP antibody, cell lysates exhibited equivalent amounts of GFP-tagged proteins (Additional file 6).

The L80F mutation dominates over L58F mutation in case of the double mutant (L58,80F), as seen by its overall effect on SAP activity (Figures 8A and 8B ). Contrary to the L58 position, L80 lies in the core of the cavity: this change in position may also explain the altered consequences of the two mutations. Overall, our results on META1 mutagenesis in its putative hydrophobic cavity are consistent with a role for META1 in secretory processes in *Leishmania *and that this cavity is important for META1's function. However, the double mutant has an altered growth (Figure 8C ), suggesting that META1 may also be participating in events other than secretion.

Overall, the consequences of META1 mutations on extracellular SAP activity are consistent in both *L. donovani *and *L. major*: L58F overexpression leads to increased SAP activity; L80F has an equivalent effect on SAP activity to WT META1 and L80F dominates over L58F in the double mutants (Additional file 8 ). Additionally, the L58F,80F double mutant has growth effects in both species (Figure 8C and Additional file 8 ). However, we did observe some differences between the two *Leishmania *species. One, the extent of effect on SAP activity is greater in *L. donovani*. Second, L80F has distinct effects in *L. major*. In *L. major*, L80F and L58,80F mutant META1 protein expression was not seen while L58F mutant protein is equivalent in expression as the overexpressed WT (Additional file 8 ). In spite of the presence of *META1-GFP *fusion transcripts in both L80F and L58,80F (Additional file 8 ), there is barely any detectable amount of mutant protein suggesting that the mutant protein is possibly getting degraded. These observations reiterate the fact that L80F mutation is dominant in L58,80F mutation. Since L80 lies in the core of the cavity which might be important for ligand binding (Additional file 7 ), it is possible that mutation at this site has disrupted ligand binding resulting in an unstable META1 conformation, leading to its degradation.

It is increasingly clear that LGT is an important mechanism in the evolution of eukaryotes [50,56]. An interesting finding has been the observed transfer of gene sets for metabolic pathways [22,57]. This is particularly of importance in the case of unicellular eukaryotes: LGT from bacteria allows the acquisition of new abilities such as exploitation of ecological niches, infective abilities and metabolic capabilities. A practical advantage of such knowledge is that such LGT acquired genes are more likely to be conducive targets for drug development since the host eukaryote typically lacks these genes [50,57]. Furthermore, over time, LGT event has played a significant role in the subsequent evolution of the *META1 *gene family in trypanosomatids, as can be seen from the occurrence of a number of paralogs in Trypanosomes and a separate gene in *Leishmania*, *META2*, which has three META domains.

It is conceivable that, for *Leishmania*, at least one of the advantages of the LGT mediated acquisition of META1 was additional modulation of secretory processes. Secretion can be an important process for a pathogen seeking to modulate its host's responses via the export of effector molecules/virulence factors. *Leishmania *secrete various bioactive molecules that are involved in pathogenesis [58]. Recently, a novel exosome-based pathway was identified as a general mechanism of protein secretion by *Leishmania *that is involved in pathogen-to-host communication and export of exosomal cargo into host macrophages [59]. Furthermore, the exosomal cargo influences myeloid cells and is immunosuppressive [60]. Our data is consistent with the correlation of META1 levels associated with *Leishmania *virulence. This work adds new information on functional role for META1 in secretory processes in *Leishmania*. Additionally, it identifies a domain within META1 that may be critical for its functions.

## Conclusions

META1 has long been associated with virulence but its activity and the processes it influences to modulate virulence have remained unknown. In order to identify the function of this enigmatic yet essential protein, we have used an evolutionary biology approach to first identify the origins of META1. Having provided several lines of evidence that *META1 *arose in *Leishmania *as the result of a LGT event of *hslJ *from bacteria, we leveraged this information to get clues about META1 function by comparing it to its bacterial homologs. We utilized 3D-modeling to show that META1 shares a structural scaffold with HslJ and MxiM. The striking structural similarity between these proteins despite a limited sequence homology points to evolutionary constraint on retaining structural folds in proteins and the retention of related functions. The sequence divergence most likely allows for specificity of interacting molecules. The conserved scaffold between META1/HslJ/MxiM identified a shared cavity in these proteins. We have extended the known involvement of MxiM in secretion and the possibility of a role for such a cavity with regards to META1 function. Using molecular biology approaches, we demonstrate that ectopic expression of META1 and site-directed mutagenesis of selected residues in the predicted META1 cavity do affect secretory processes in *Leishmania*, thus establishing a function for this poorly characterized protein as well as identifying a functional domain within the molecule. Overall, our study demonstrates the utility of a multidisciplinary approach that combines insights from evolutionary origins of a molecule with bioinformatics analyses and computational approaches, leading to a predicted structure and activity, both of which are testable at a molecular level.

## Methods

### Sequences, sequence similarity searches and sequence alignment

BLASTP and PSI-BLAST searches [61] were performed against the NRDB using the NCBI server http://www.ncbi.nlm.nih.gov/BLAST. Pfam search [62] was performed using HMMer via the Pfam server at http://pfam.sanger.ac.uk/. All the sequences (DNA/protein) used in the study for codon bias, phylogenetic analysis and synonymous & non-synonymous rate estimation were obtained from NCBI. For *E. coli *HslJ, sequence information was used from accession number NP_4158971.1. Sequence alignment was done with CLUSTALW available at http://www.ebi.ac.uk/clustalw/ or EMBOSS http://www.ebi.ac.uk/Tools/emboss/align/index.html. Sequence to structure alignment was done with PROMALS3D software available at http://prodata.swmed.edu/promals3d/promals3d.php[63].

### GC content

Total GC content and GC content at codon positions for all *L. major *ORFs and *META1 *was obtained using Integrated Genomic Island Prediction Tool (IGIPT) software available at http://ccnsb.iiit.ac.in/nita/IGIPT/srk/index.php[64]. FASTA files for all *L. major *ORFs was obtained from the Wellcome Trust Sanger Institute at ftp://ftp.sanger.ac.uk/pub/databases/L.major_sequences/.

### Codon usage bias

For calculating Codon Adaptation Index (CAI), the Relative Synonymous Codon Usage (RSCU) values of the highly expressed genes of *L. major *were obtained from [65]. CAI values of reference genes from Additional file 2 and *META1 *were calculated manually as described earlier [66].

### Phylogenetic analysis

The sequences were aligned using MUSCLE [67] on default settings. Gaps were removed from the alignment manually. Phylogenetic tree was generated by PhyML [68] using the evolutionary model WAG, the gamma-distribution model, four rate categories and invariant position. The WAG model was found to be the best-fitting model using PROTTEST [69]. The gamma parameter and the fraction of invariant positions were estimated from the data. The trees were optimized for topology, branch length and rate parameters. Each tree was subjected to 100 bootstrap replicates. iTOL [70] was used for viewing the phylogenetic tree and later on modified in Adobe Photoshop CS4 for publication. Re-rooting of the tree was also done using iTOL. The accession numbers of the protein sequences used are given in Additional file 11.

### Estimation of substitution rates

Synonymous (K_s_) and non-synonymous (K_a_) substitution rates were estimated using the methods as described earlier [23] as implemented in yn00 in the PAML software [71].

### Molecular modeling of *Leishmania *META1

Consensus structure prediction for META1 was performed using 3D-Jury http://bioinfo.pl/meta; [29]). MODELLER 9v2 [30] software was used to obtain the final model of META1 based on the NMR structure of an *E. coli *HslJ (PDB code 2kts). The model was viewed using the program O [72] and was compared with that of the template and related structures. The program O was also used for superposition of META1 and HslJ structures on that of *S. flexneri *secretin pilot protein, MxiM (PDB code 1y9t) for comparison. The figures were made using PyMol [73].

### *Leishmania *strains and growth condition

*L. donovani *MHOM/IN/1983/AG83 and *L. major *MHOM/Su73/5ASKH were used for all the experiments. The conditions for promastigotes *Leishmania *cultures are as described earlier [74]. For all experiments, cell numbers were estimated by direct counting in a hemocytometer under a light microscope. The promastigotes were routinely inoculated at a starting density of 10^6^cells/ml; typically, logarithmic growth was observed between day 2-4 and stationary phase between day 5 and 7. Hence, in all experiments, 3 days and 7 days old cultures were used as representative of log phase and stationary phase respectively. For axenic amastigotes, log phase promastigotes were pelleted down by centrifugation at 3000 rpm for 10 min, washed with PBS and resuspended in amastigote media (Complete HOMEM, 20% FCS, pH 5.5) and incubated at 37°C with 5% CO_2 _for 48 hrs_. _Promastigotes convert into amastigotes within 48 hrs. For generating attenuated parasite line, virulent promastigotes were passaged in HOMEM medium once a week until 30 passages and thereafter, infectivity of the parasites were checked in BALB/c mice after 4 weeks of inoculation.

### RNA isolation, reverse transcription and quantitative real time (QRT) PCR analysis

Total RNA was isolated using the acid guanidinium isothiocyanate method [75]. 1 μg of total RNA from *L. donovani *was treated with DNAse I (Ambion Inc.). Synthesis of cDNA was performed by using First Strand Synthesis kit and the Superscript III Reverse Transcriptase (Invitrogen), according to the manufacturer's instructions. All real-time PCR experiments were performed in ABI prism 7900 HT sequence detection system (ABI) as described earlier [76]. The PCR conditions were as follows: 95°C for 10 min, 95°C for 15 sec, 58°C for 30 sec and 72°C for 30 sec for 40 cycles. Following primers were used in QRT-PCR analysis; for *LdMETA1*: TV359 & TV360, for *LmjMETA1*: TV672 & TV758, for neomycin: TV 397 & TV398, for GFP: TV276 & TV396 and for *GAPDH*: TV366 & TV367. Sequences of all the primers are mentioned in Additional file 12. All the reactions were analyzed using the software (SDS 2.3) provided with the instrument. The relative expression of the genes was calculated by using 2^-ΔΔCt ^formula using *GAPDH *as a normalizer. The values reported are the mean of at least three biological replicates. The standard deviation from the mean is shown as error bars in each group.

### Expression and purification of recombinant *Ld*META1 protein and generation of antibodies

The *Ld*META1 coding region was PCR amplified from *L. donovani *genomic DNA, using primers TV213 & TV215 and cloned in *EcoRI-HindIII *site of pET28a+ vector (Novagen). Primer sequences are listed in Additional file 12. The recombinant constructs were transformed in *E. coli *BL21 (DE3) strain and protein expression was induced at A_600 _of 0.6 with 1 mM of isopropyl-1-β-D-galactopyranoside at 37 °C for 3 hours. Recombinant 6X-His-META1 protein was purified from bacterial cells in native conditions using the Ni^2+^-nitrilo triacetic acid agarose resin (Qiagen), according to the manufacturer's instructions.

For raising polyclonal antibodies against recombinant 6X-His-META1 protein in the mouse, 50 μg/animal of protein was mixed with an equal volume of Freund's complete adjuvant. The mixture was made into an emulsion by passing through a 2 ml syringe (1.5 inch 19G needle) and intermittently keeping it at 4°C. This emulsion was injected into the mouse subcutaneously. After 14 days, the first booster dose was given in a similar manner, except that Freund's complete adjuvant was replaced with Freund's incomplete adjuvant. Subsequent booster doses were given after a 14-day interval and the antibody titer was checked using dot blot. For determination of antibody titer 20 ng of purified protein was spotted in a row, air-dried and incubated with different concentrations of immune serum (1:1,000 to 1:5,000). Western blot experiments were carried out when the appropriate titer (1:5,000) was obtained following booster doses. Mice were sacrificed and blood collected from the inferior vena cava using a 23G needle and a 2 ml syringe. The blood was allowed to stand at room temperature for a couple of hours and then kept at 4°C for overnight to allow formation of a firm clot. Red blood cells were removed by centrifugation, the sera were collected in a fresh tube, 0.2% NaN_3 _was added and stored at 4°C. All experiments involving animals were performed in compliance with recommendations and permission of the CCMB Institutional Animal Ethics Committee.

### Generation of pX63-*META1::GFP *constructs

The *META1 *gene of both *L. donovani *and *L. major *were cloned in frame with *GFP *at the C-terminus and further this *META1-GFP *cassette was ligated into *BamHI-XbaI *site of pX63-*NEO *vector. The *L. donovani *and *L. major META1 *genes were amplified from genomic DNA by using the primer combinations TV494 & TV757 and TV494 & TV758 respectively and *GFP *was amplified using TV276 & TV277 from pX63-*EGFP *vector. PCR amplicons of *META1 *gene of both *L. donovani *&*L. major *and *GFP *were first cloned using pMOS blunt end cloning vector kit (GE Healthcare) as per manufacturer's instructions. Inserts were released from pMOS by using restriction enzymes inserted in the primer sequences. Extra sequences were added in the primers to maintain the reading frame and/or to add restriction sites for cloning: for *META1*, TV494 with a *BamHI *and TV757 & TV758 with *NcoI *site respectively and for *GFP*, TV276 & TV277 with an *NcoI *and *XbaI *site respectively. Primer sequences are listed in Additional file 12.

### Transfection of plasmids into *Leishmania *promastigotes

*Leishmania *promastigotes were grown to late log phase (~2 × 10^7^cells/ml). Transfection protocol is the same as described earlier [77]. Briefly, cells were washed and resuspended in ice cold electroporation buffer (137 mM NaCl, 5 mM KCl, 0.7 mM Na_2_HPO_4_, 6 mM glucose, 21 mM HEPES pH 7.5) at a density of 10^8 ^cells/ml. 400 μl of cells and 100 μg of plasmid DNA were mixed well into 0.2 mm cuvettes (Biorad) then electroporated in Genepulser II apparatus (Bio-Rad Laboratories) with the pulse of 450 volts (2.25 V/cm) and capacitance 500 μF. Cuvettes were immediately placed on ice for 10 minutes. Cells were transferred to 5 ml of complete HOMEM media. After 48 hours, transformants were selected in HOMEM containing G418 until the control, untransfected cells died completely in the presence of the antibiotic. Concentrations of G418 used were: 40 μg/ml for virulent *L. donovani *&*L. major *promastigotes and 200 μg/ml for attenuated *L. donovani *promastigotes. For further experiments, pool populations were maintained in liquid media containing G418 in above mentioned concentrations for the respective cell types.

### Site-directed mutagenesis

For generating constructs with point mutations in *META1*, site-directed mutagenesis (SDM) was carried out using QuickChange-XL kit from Stratagene as per manufacturer's instructions. pX63-*LdMETA1::GFP *(*Ld/Ld-*WT) and pX63-*LmjMETA1::GFP *(*Lmj/Lmj-*WT) were used as templates for SDM of *L. donovani *and *L. major META1 *mutagenesis. Details of the primers used are given in Additional file 12.

### Western blot analysis

*Leishmania *promastigotes or amastigotes at different stages of growth were harvested, washed in PBS and then resuspended in 1X-PBS with 1X-protease inhibitor cocktail (Roche) and then sonicated using VibraCell (Sonics & Materials Inc.) with 50% amplitude and three pulses for 30 sec each. 30 μg of protein lysate from each sample were separated on 15% SDS-PAGE and transferred onto nitrocellulose Hybond ECL membranes (Amersham Biosciences). Membranes were blocked with 5% non-fat dried milk in 1X-TBS-T (10 mM Tris-HCl, pH 8.0, 150 mM NaCl, 0.1% Tween-20). The following antibodies were used: mouse anti-*Ld*META1 (1:5,000) was raised against recombinant N-terminally 6X-His tagged *Ld*META1 protein, rabbit polyclonal BiP antisera (1:15,000), rabbit polyclonal GFP antibody (Abcam; 1:4000), sheep polyclonal GP63 antisera (1:10,000), anti-mouse or anti rabbit biotinylated antibodies (1:5,000, Amersham), avidin conjugated with horseradish peroxidase (1:10,000, Amersham Biosciences) and anti-sheep conjugated with horseradish peroxidase (1:5,000, Santa Cruz Biotechnology). After incubation, membranes were washed three times with 1X-TBS-T. Immunodetection was carried out using the ECL western blotting detection system (GE Healthcare) according to the manufacturer's instructions and images were obtained using BIOMAX™-XBT X-ray film (Kodak). Images of western blots were quantified by ImageJ software available at http://rsbweb.nih.gov/ij/[78].

For western blots on the culture supernatants, media from cultures were passed through a 0.22 μm membrane and concentrated 50-fold using Millipore Amicon 5 kDa cut-off and loaded onto SDS-PAGE gel along with whole cell lysates as control.

### Secreted acid phosphatase (SAP) assay

SAP activity was determined in culture supernatants as previously described [41]. After seeding cells at a density of 10^6^cells/ml, aliquots of culture medium were removed at log and stationary phase, filtered through 0.22 μm pores membrane to remove cells and debris. 138 μl supernatant was then incubated in a final volume of 200 μl with 50 mM para-nitrophenyl phosphate (pNPP), 50 mM sodium acetate pH 4 and 0.1% β-mercaptoethanol (v/v) for 30 minutes at 37 °C. The reaction was stopped with 800 μl of 2M sodium hydroxide and the absorbance of the released para-nitrophenol was determined at 410 nm. For intracellular SAP activity, cell pellets were washed with PBS and were lysed on ice for 30 min at 10^8 ^cells/ml in 50 mM acetate buffer and 1% Triton X-100. 100 μl of lysate was then used for SAP assay with 50 mM pNPP.

## Competing interests

The authors declare that they have no competing interests.

## Authors' contributions

VP participated in design of the study, carried out bioinformatics analysis, laboratory experiments, analyzed the data and drafted the manuscript. AG and RS constructed the homology model and analyzed the structures. AJE helped design the analysis of evolutionary origin of META1, participated in data analysis and critically read the manuscript. TV participated in design of the study, supervised the work, analyzed the data and drafted the manuscript. All authors read and approved the final manuscript.

## Supplementary Material

Additional file 1 **List of protein sequences obtained from BLASTP sequence similarity search for *Lmj*META1 against non-redundant NCBI database**. Table S1. Description of BLASTP hits with their e-value. Click here for file

Additional file 2 **List of reference genes for CAI determination**. Table S2. Description of reference genes used for CAI determination. Click here for file

Additional file 3 **Codon Adaptation Index (CAI) of *META1 *homologs in *T. cruzi***. Figure S1. CAI of 2 *META1 *homologs in *T. cruzi *compared with RP S8 and α-Tubulin. Click here for file

Additional file 4 **Quantitation of signal in western blots in **Figure 5. Figure S2. Images of western blots in Figure 5B, C and 5D were quantitated by ImageJ software. Click here for file

Additional file 5 **Movie of superposed structures of HslJ (magenta), MxiM (yellow) and META1 model (green)**. Figure S3. Click here for file

Additional file 6 **Overexpression of META1 in *L. donovani***. Figure S4. Western blots (S4A and S4B) of *L. donovani *virulent and attenuated META1 transfectants with META1, BiP and GFP antibodies. S4C represents effect of META1 overexpression on growth kinetics of virulent and attenuated *L. donovani *compared to their respective wild-types. Click here for file

Additional file 7 **Mutagenesis of META1**. Figure S5. Sequence alignment of META1 homologs and stereoscopic representation of META1 model highlighting the leucine 58 and leucine 80. Click here for file

Additional file 8 **Effect of META1 overexpression in *L. major*. **Figure S6. Effect of WT and mutant META1 overexpression in *L. major *on extracellular SAP activity (S6A) and growth kinetics (S6B). S6C and S6D represents western blot and QRT-PCR of *L. major *META1 transfectants respectively. Click here for file

Additional file 9 **Western blot of *L. donovani *whole cell lysates v/s culture supernatants**. Figure S7. Western blot with GP63 and BiP on whole cell lysates and culture supernatants of *L. donovani *META1 transfectants. Click here for file

Additional file 10 **Phylogenetic tree re-rooted at two different roots**. Figure S8. Phylogenetic tree representation generated in Figure 3 was re-rooted at *Tolumonas *(S8A) and *T. cruzi *Hypoth3 (S8B). Click here for file

Additional file 11 **List of protein sequences used in phylogenetic analysis**. Table S3. Accession numbers of protein sequences. Click here for file

Additional file 12 **Primers used in the study**. Table S4. List of sequences of all the primers used in this study. Click here for file

## References

[B1] Brittingham A Morrison CJ McMaster WR McGwire BS Chang KP Mosser DM Role of the *Leishmania *surface protease gp63 in complement fixation, cell adhesion, and resistance to complement-mediated lysis J Immunol 1995 155 3102 3111 7673725

[B2] Chakrabarty R Mukherjee S Lu HG McGwire BS Chang KP Basu MK Kinetics of entry of virulent and avirulent strains of *Leishmania donovani *into macrophages: a possible role of virulence molecules (gp63 and LPG) J Parasitol 1996 82 632 635 10.2307/3283790 8691373

[B3] Joshi PB Kelly BL Kamhawi S Sacks DL McMaster WR Targeted gene deletion in *Leishmania major *identifies leishmanolysin (GP63) as a virulence factor Mol Biochem Parasitol 2002 120 33 40 10.1016/S0166-6851(01)00432-7 11849703

[B4] McGwire BS O'Connell WA Chang KP Engman DM Extracellular release of the glycosylphosphatidylinositol (GPI)-linked *Leishmania *surface metalloprotease, gp63, is independent of GPI phospholipolysis: implications for parasite virulence J Biol Chem 2002 277 8802 8809 10.1074/jbc.M109072200 11777912

[B5] Yao C Donelson JE Wilson ME The major surface protease (MSP or GP63) of *Leishmania *sp. Biosynthesis, regulation of expression, and function Mol Biochem Parasitol 2003 132 1 16 10.1016/S0166-6851(03)00211-1 14563532

[B6] Descoteaux A Turco SJ The lipophosphoglycan of *Leishmania *and macrophage protein kinase C Parasitol Today 1993 9 468 471 10.1016/0169-4758(93)90105-O 15463696

[B7] Beverley SM Turco SJ Lipophosphoglycan (LPG) and the identification of virulence genes in the protozoan parasite *Leishmania* Trends Microbiol 1998 6 35 40 10.1016/S0966-842X(97)01180-3 9481823

[B8] Spath GF Epstein L Leader B Singer SM Avila HA Turco SJ Lipophosphoglycan is a virulence factor distinct from related glycoconjugates in the protozoan parasite *Leishmania major* Proc Natl Acad Sci USA 2000 97 9258 9263 10.1073/pnas.160257897 10908670 PMC16855

[B9] Garami A Mehlert A Ilg T Glycosylation defects and virulence phenotypes of *Leishmania mexicana *phosphomannomutase and dolicholphosphate-mannose synthase gene deletion mutants Mol Cell Biol 2001 21 8168 8183 10.1128/MCB.21.23.8168-8183.2001 11689705 PMC99981

[B10] Descoteaux A Avila HA Zhang K Turco SJ Beverley SM *Leishmania *LPG3 encodes a GRP94 homolog required for phosphoglycan synthesis implicated in parasite virulence but not viability EMBO J 2002 21 4458 4469 10.1093/emboj/cdf447 12198148 PMC126187

[B11] Spath GF Garraway LA Turco SJ Beverley SM The role(s) of lipophosphoglycan (LPG) in the establishment of *Leishmania major *infections in mammalian hosts Proc Natl Acad Sci USA 2003 100 9536 9541 10.1073/pnas.1530604100 12869694 PMC170953

[B12] Gaur U Showalter M Hickerson S Dalvi R Turco SJ Wilson ME *Leishmania donovani *lacking the Golgi GDP-Man transporter LPG2 exhibit attenuated virulence in mammalian hosts Exp Parasitol 2009 122 182 191 10.1016/j.exppara.2009.03.014 19328787 PMC2720449

[B13] Pollock KG McNeil KS Mottram JC Lyons RE Brewer JM Scott P The *Leishmania mexicana *cysteine protease, CPB2.8, induces potent Th2 responses J Immunol 2003 170 1746 1753 12574338 10.4049/jimmunol.170.4.1746

[B14] Buxbaum LU Denise H Coombs GH Alexander J Mottram JC Scott P Cysteine protease B of *Leishmania mexicana *inhibits host Th1 responses and protective immunity J Immunol 2003 171 3711 3717 14500670 10.4049/jimmunol.171.7.3711

[B15] Mahmoudzadeh-Niknam H McKerrow JH *Leishmania tropica*: cysteine proteases are essential for growth and pathogenicity Exp Parasitol 2004 106 158 163 10.1016/j.exppara.2004.03.005 15172223

[B16] Nourbakhsh F Uliana SR Smith DF Characterisation and expression of a stage-regulated gene of *Leishmania major* Mol Biochem Parasitol 1996 76 201 213 10.1016/0166-6851(95)02559-6 8920007

[B17] Uliana SR Goyal N Freymuller E Smith DF *Leishmania*: overexpression and comparative structural analysis of the stage-regulated *meta 1 *gene Exp Parasitol 1999 92 183 191 10.1006/expr.1999.4410 10403759

[B18] Ramos CS Franco FA Smith DF Uliana SR Characterisation of a new *Leishmania *META gene and genomic analysis of the META cluster FEMS Microbiol Lett 2004 238 213 219 15336424 10.1016/j.femsle.2004.07.037

[B19] Lawrence JG Ochman H Amelioration of bacterial genomes: rates of change and exchange J Mol Evol 1997 44 383 397 10.1007/PL00006158 9089078

[B20] Lawrence JG Ochman H Molecular archaeology of the *Escherichia coli *genome Proc Natl Acad Sci USA 1998 95 9413 9417 10.1073/pnas.95.16.9413 9689094 PMC21352

[B21] Mrazek J Karlin S Detecting alien genes in bacterial genomes Ann N Y Acad Sci 1999 870 314 329 10.1111/j.1749-6632.1999.tb08893.x 10415493

[B22] Opperdoes FR Michels PA Horizontal gene transfer in trypanosomatids Trends Parasitol 2007 23 470 476 10.1016/j.pt.2007.08.002 17826337

[B23] Yang Z Nielsen R Estimating synonymous and nonsynonymous substitution rates under realistic evolutionary models Mol Biol Evol 2000 17 32 43 10666704 10.1093/oxfordjournals.molbev.a026236

[B24] Dowd SE Ishizaki H Microarray based comparison of two *Escherichia coli *O157:H7 lineages BMC Microbiol 2006 6 30 10.1186/1471-2180-6-30 16539702 PMC1431545

[B25] Chuang SE Blattner FR Characterization of twenty-six new heat shock genes of *Escherichia coli* J Bacteriol 1993 175 5242 5252 8349564 10.1128/jb.175.16.5242-5252.1993 PMC204992

[B26] Saar Y Ransford A Waldman E Mazareb S min-Spector S Plumblee J Characterization of developmentally-regulated activities in axenic amastigotes of *Leishmania donovani* Mol Biochem Parasitol 1998 95 9 20 10.1016/S0166-6851(98)00062-0 9763285

[B27] Debrabant A Joshi MB Pimenta PF Dwyer DM Generation of *Leishmania donovani *axenic amastigotes: their growth and biological characteristics Int J Parasitol 2004 34 205 217 10.1016/j.ijpara.2003.10.011 15037106

[B28] Alcolea PJ Alonso A Gomez MJ Sanchez-Gorostiaga A Moreno-Paz M Gonzalez-Pastor E Temperature increase prevails over acidification in gene expression modulation of amastigote differentiation in *Leishmania infantum* BMC Genomics 2010 11 31 10.1186/1471-2164-11-31 20074347 PMC2845110

[B29] Ginalski K Rychlewski L Protein structure prediction of CASP5 comparative modeling and fold recognition targets using consensus alignment approach and 3D assessment Proteins 2003 53 Suppl 6 410 417 14579329 10.1002/prot.10548

[B30] Sali A Blundell TL Comparative protein modelling by satisfaction of spatial restraints J Mol Biol 1993 234 779 815 10.1006/jmbi.1993.1626 8254673

[B31] Holm L Rosenström P Dali server: conservation mapping in 3D Nucl Acids Res 2010 38 545 549 10.1093/nar/gkq366 PMC2896194 20457744

[B32] Hasegawa H Holm L Advances and pitfalls of protein structural alignment Curr Opin Struct Biol 2009 19 341 348 10.1016/j.sbi.2009.04.003 19481444

[B33] Schuch R Maurelli AT MxiM and MxiJ, base elements of the Mxi-Spa type III secretion system of *Shigella*, interact with and stabilize the MxiD secretin in the cell envelope J Bacteriol 2001 183 6991 6998 10.1128/JB.183.24.6991-6998.2001 11717255 PMC95545

[B34] Schuch R Maurelli AT The mxi-Spa type III secretory pathway of *Shigella flexneri *requires an outer membrane lipoprotein, MxiM, for invasin translocation Infect Immun 1999 67 1982 1991 10085046 10.1128/iai.67.4.1982-1991.1999 PMC96556

[B35] Lario PI Pfuetzner RA Frey EA Creagh L Haynes C Maurelli AT Structure and biochemical analysis of a secretin pilot protein EMBO J 2005 24 1111 1121 10.1038/sj.emboj.7600610 15775974 PMC556411

[B36] Overath P Stierhof YD Wiese M Endocytosis and secretion in trypanosomatid parasites - Tumultuous traffic in a pocket Trends Cell Biol 1997 7 27 33 10.1016/S0962-8924(97)10046-0 17708895

[B37] Grzyb J Latowski D Strzalka K Lipocalins - a family portrait J Plant Physiol 2006 163 895 915 10.1016/j.jplph.2005.12.007 16504339

[B38] Bates PA Dwyer DM Biosynthesis and secretion of acid phosphatase by *Leishmania donovani *promastigotes Mol Biochem Parasitol 1987 26 289 296 10.1016/0166-6851(87)90081-8 3323906

[B39] Bates PA Hermes I Dwyer DM *Leishmania donovani*: immunochemical localization and secretory mechanism of soluble acid phosphatase Exp Parasitol 1989 68 335 346 10.1016/0014-4894(89)90115-X 2649391

[B40] Bates PA Hermes I Dwyer DM Golgi-mediated post-translational processing of secretory acid phosphatase by *Leishmania donovani *promastigotes Mol Biochem Parasitol 1990 39 247 255 10.1016/0166-6851(90)90063-R 2320058

[B41] Cuvillier A Redon F Antoine JC Chardin P DeVos T Merlin G LdARL-3A, a *Leishmania *promastigote-specific ADP-ribosylation factor-like protein, is essential for flagellum integrity J Cell Sci 2000 113 Pt 11 2065 2074 10806117 10.1242/jcs.113.11.2065

[B42] Parodi-Talice A Araújo JM Torres C Pérez-Victoria JM Gamarro F Castanys S The overexpression of a new ABC transporter in *Leishmania *is related to phospholipid trafficking and reduced infectivity Biochim Biophys Acta 2003 1612 195 207 10.1016/S0005-2736(03)00131-7 12787938

[B43] Sahin A Espiau B Tetaud E Cuvillier A Lartigue L Ambit A Robinson DR Merlin G The *Leishmania *ARL-1 and Golgi traffic PLoS ONE 2008 3 e1620 10.1371/journal.pone.0001620 18286177 PMC2237903

[B44] Nara T Hshimoto T Aoki T Evolutionary implications of the mosaic pyrimidine-biosynthetic pathway in eukaryotes Gene 2000 257 209 222 10.1016/S0378-1119(00)00411-X 11080587

[B45] Molinas SM Altabe SG Opperdoes FR Rider MH Michels PA Uttaro AD The multifunctionalisopropyl alcohol dehydrogenase of *Phytomonas *sp. could be the result of a horizontal gene transfer from a bacterium to the trypanosomatid lineage J Biol Chem 2003 278 36169 36175 10.1074/jbc.M305666200 12853449

[B46] Cazalet C Rusniok C Bruggemann H Zidane N Magnier A Ma L Evidence in the *Legionella pneumophila *genome for exploitation of host cell functions and high genome plasticity Nat Genet 2004 36 1165 1173 10.1038/ng1447 15467720

[B47] Chien M Morozova I Shi S Sheng H Chen J Gomez SM The genomic sequence of the accidental pathogen *Legionella pneumophila* Science 2004 305 1966 1968 10.1126/science.1099776 15448271

[B48] de Felipe KS Pampou S Jovanovic OS Pericone CD Ye SF Kalachikov S Evidence for acquisition of *Legionella *type IV secretion substrates via interdomain horizontal gene transfer J Bacteriol 2005 187 7716 7726 10.1128/JB.187.22.7716-7726.2005 16267296 PMC1280299

[B49] Annoura T Nara T Makiuchi T Hashimoto T Aoki T The origin of dihydroorotate dehydrogenase genes of kinetoplastids, with special reference to their biological significance and adaptation to anaerobic, parasitic conditions J Mol Evol 2005 60 113 127 10.1007/s00239-004-0078-8 15696374

[B50] Whitaker JW McConkey GA Westhead DR Prediction of horizontal gene transfers in eukaryotes: approaches and challenges Biochem Soc Trans 2009 37 792 5 10.1042/BST0370792 19614596

[B51] Kobayashi H Miyamoto T Hashimoto Y Kiriki M Motomatsu A Honjoh K Identification of factors involved in recovery of heat-injured *Salmonella Enteritidis* J Food Prot 2005 68 932 941 15895724 10.4315/0362-028x-68.5.932

[B52] Lilic M Jovanovic M Jovanovic G Savic DJ Identification of the CysB-regulated gene, hslJ, related to the *Escherichia coli *novobiocin resistance phenotype FEMS Microbiol Lett 2003 224 239 246 10.1016/S0378-1097(03)00441-5 12892888

[B53] Ramos CS Yokoyama-Yasunaka JK Guerra-Giraldez C Price HP Mortara RA Smith DF *Leishmania amazonensis *META2 protein confers protection against heat shock and oxidative stress Exp Parasitol 2011 127 228 37 10.1016/j.exppara.2010.08.004 20713053

[B54] Luo Y Bertero MG Frey EA Pfuetzner RA Wenk MR Creagh L Structural and biochemical characterization of the type III secretion chaperones CesT and SigE Nat Struct Biol 2001 8 1031 1036 10.1038/nsb717 11685226

[B55] Birtalan SC Phillips RM Ghosh P Three-dimensional secretion signals in chaperone-effector complexes of bacterial pathogens Mol Cell 2002 9 971 980 10.1016/S1097-2765(02)00529-4 12049734

[B56] Keeling PJ Palmer JD Horizontal gene transfer in eukaryotic evolution Nat Rev Genet 2008 9 605 10.1038/nrg2386 18591983

[B57] Whitaker JW McConkey GA Westhead DR The transferome of metabolic genes explored: analysis of the horizontal transfer of enzyme encoding genes in unicellular eukaryotes Genome Biol 2009 10 R36 10.1186/gb-2009-10-4-r36 19368726 PMC2688927

[B58] Wilson ME Jeronimo SM Pearson RD Immunopathogenesis of infection with the visceralizing *Leishmania *species Microb Pathog 2005 38 147 160 10.1016/j.micpath.2004.11.002 15797810

[B59] Silverman JM Clos J de'Oliveira CC Shirvani O Fang Y Wang C An exosome-based secretion pathway is responsible for protein export from *Leishmania *and communication with macrophages J Cell Sci 2010 123 842 852 10.1242/jcs.056465 20159964

[B60] Silverman JM Clos J Horakova E Wang AY Wiesgigl M Kelly I *Leishmania *exosomes modulate innate and adaptive immune responses through effects on monocytes and dendritic cells J Immunol 2010 185 5011 5022 10.4049/jimmunol.1000541 20881185

[B61] Altschul SF Madden TL Schaffer AA Zhang J Zhang Z Miller W Gapped BLAST and PSI-BLAST: a new generation of protein database search programs Nucleic Acids Res 1997 25 3389 3402 10.1093/nar/25.17.3389 9254694 PMC146917

[B62] Finn RD Mistry J Tate J Coggill P Heger A Pollington JE The Pfam protein families database Nucleic Acids Res 2010 38 D211 D222 10.1093/nar/gkp985 19920124 PMC2808889

[B63] Pei J Kim BH Grishin NV PROMALS3D: a tool for multiple protein sequence and structure alignments Nucleic Acids Res 2008 36 2295 2300 10.1093/nar/gkn072 18287115 PMC2367709

[B64] Jain R Ramineni S Parekh N Integrated Genomic Island Prediction Tool (IGIPT) IEEE Proceedings of International Conference on Information Technology (ICIT2008), icit 2008 131 132

[B65] Chanda I Pan A Saha SK Dutta C Comparative codon and amino acid composition analysis of Tritryps-conspicuous features of *Leishmania major* FEBS Lett 2007 581 5751 5758 10.1016/j.febslet.2007.11.041 18037385

[B66] Sharp PM Li WH The Codon Adaptation Index--a measure of directional synonymous codon usage bias, and its potential applications Nucleic Acids Res 1987 15 1281 1295 10.1093/nar/15.3.1281 3547335 PMC340524

[B67] Edgar RC MUSCLE: multiple sequence alignment with high accuracy and high throughput Nucleic Acids Res 2004 32 1792 1797 10.1093/nar/gkh340 15034147 PMC390337

[B68] Guindon S Dufayard JF Lefort V Anisimova M Hordijk W Gascuel O New Algorithms and Methods to Estimate Maximum-Likelihood Phylogenies: Assessing the Performance of PhyML 3.0 Systematic Biology 2010 59 307 21 10.1093/sysbio/syq010 20525638

[B69] Abascal F Zardoya R Posada D ProtTest: Selection of best-fit models of protein evolution Bioinformatics 2005 21 2104 2105 10.1093/bioinformatics/bti263 15647292

[B70] Letunic I Bork P Interactive Tree Of Life v2: online annotation and display of phylogenetic trees made easy Nucleic Acids Res 2011 10.1093/nar/gkr201 PMC3125724 21470960

[B71] Yang Z PAML: a program package for phylogenetic analysis by maximum likelihood Comput Appl Biosci 1997 13 555 556 9367129 10.1093/bioinformatics/13.5.555

[B72] Jones TA Zou JY Cowan SW Kjeldgaard M Improved methods for building protein models in electron density maps and the location of errors in these models Acta Crystallogr A 1991 47 Pt 2 110 119 2025413 10.1107/s0108767390010224

[B73] DeLano WL The PyMol molecular graphics system 2004 San Carlos, CA: DeLano Scientific LLC

[B74] Bera A Singh S Nagaraj R Vaidya T Induction of autophagic cell death in *Leishmania donovani *by antimicrobial peptides Mol Biochem Parasitol 2003 127 23 35 10.1016/S0166-6851(02)00300-6 12615333

[B75] Chomczynski P Sacchi N The single-step method of RNA isolation by acid guanidinium thiocyanate-phenol-chloroform extraction: twenty-something years on Nat Protoc 2006 1 581 585 10.1038/nprot.2006.83 17406285

[B76] Tupperwar N Vineeth V Rath S Vaidya T Development of a real-time polymerase chain reaction assay for the quantification of *Leishmania *species and the monitoring of systemic distribution of the pathogen Diagn Microbiol Infect Dis 2008 61 23 30 10.1016/j.diagmicrobio.2007.12.013 18255247

[B77] Kapler GM Coburn CM Beverley SM Stable transfection of the human parasite *Leishmania major *delineates a 30-kilobase region sufficient for extrachromosomal replication and expression Mol Cell Biol 1990 10 1084 1094 2304458 10.1128/mcb.10.3.1084-1094.1990 PMC360971

[B78] Rasband WS ImageJ 1997 US National Institutes of Health, Bethesda, Maryland, USA http://imagej.nih.gov/ij/

