# Supplementary material for: Evolutionary and functional insights into Leishmania META1: evidence for lateral gene transfer and a role for META1 in secretion
Source: BMC Evol Biol. 2011 Nov 17;11:334. doi: 10.1186/1471-2148-11-334 (PMC3270026; doi:10.1186/1471-2148-11-334)
Supplement: Additional file 1 — List of protein sequences obtained from BLASTP sequence similarity search for LmjMETA1 against non-redundant NCBI database. Table S1. Description of BLASTP hits with their e-value. [file 1471-2148-11-334-S1.PDF]

**Table S1.** List of protein sequences obtained from BLASTP sequence similarity search for *Lmj*META1 against non-redundant NCBI database

| Species Name | Description | Accession | E-value |
|---|---|---|---|
| *Leishmania major* | Infective insect stage-specific protein | XP_001682347.1 | $1e^{-57}$ |
| *Leishmania infantum* | Hypothetical protein | XP_001464758.1 | $5e^{-52}$ |
| *Leishmania donovani* | Infective insect stage-specific protein | AAC04778.1 | $5e^{-52}$ |
| *Leishmania amazonensis* | META1 protein | AAC04758.1 | $4e^{-46}$ |
| *Leishmania braziliensis* | Hypothetical protein | XP_001563865.1 | $3e^{-29}$ |
| *Trypanosoma brucei TREU927* | Hypothetical protein | XP_844890.1 | $2e^{-9}$ |
| *Trypanosoma brucei TREU927* | Hypothetical protein | XP_844894.1 | $7e^{-8}$ |
| *Trypanosoma brucei TREU927* | Hypothetical protein | XP_844891.1 | $1e^{-7}$ |
| *Trypanosoma brucei TREU927* | Hypothetical protein | XP_844900.1 | $1e^{-6}$ |
| *Leishmania infantum* | Hypothetical protein | XP_001464756.1 | $4e^{-6}$ |
| *Leishmania braziliensis* | Hypothetical protein | XP_001563874.1 | $5e^{-6}$ |
| *Leishmania major* | Hypothetical protein | XP_001682345.1 | $6e^{-6}$ |
| *Leishmania amazonensis* | META2 protein | AAL32493.2 | $4e^{-5}$ |
| *Trypanosoma brucei TREU927* | Hypothetical protein | XP_844893.1 | $9e^{-5}$ |
| *Trypanosoma cruzi strain CL Brener* | Hypothetical protein | XP_814398.1 | $1e^{-4}$ |
