# Supplementary material for: Evolutionary and functional insights into Leishmania META1: evidence for lateral gene transfer and a role for META1 in secretion
Source: BMC Evol Biol. 2011 Nov 17;11:334. doi: 10.1186/1471-2148-11-334 (PMC3270026; doi:10.1186/1471-2148-11-334)
Supplement: Additional file 2 — List of reference genes for CAI determination. Table S2. Description of reference genes used for CAI determination. [file 1471-2148-11-334-S2.PDF]

Table S2. List of reference genes for CAI determination

Gene Name	Gene ID	Description	No. of Codons
Amastin	gi|2660761:6-557	Specific to Tritryps	183
GP63	gi|157865342	Specific to Tritryps	602
Ribosomal Protein S8	gi|157870350	Generic	220
Hexokinase	gi|54292804	Generic	471
α- Tubulin	gi|157866135	Eukaryotic	451
PTR-1	gi|157869600	Predicted LGT from bacteria	288
Coproporphyrinogen	LmjF06.1270	Predicted LGT from bacteria	301
