# Supplementary material for: Evolutionary and functional insights into Leishmania META1: evidence for lateral gene transfer and a role for META1 in secretion
Source: BMC Evol Biol. 2011 Nov 17;11:334. doi: 10.1186/1471-2148-11-334 (PMC3270026; doi:10.1186/1471-2148-11-334)
Supplement: Additional file 3 — Codon Adaptation Index (CAI) of META1 homologs in T. cruzi. Figure S1. CAI of 2 META1 homologs in T. cruzi compared with RP S8 and α-Tubulin. [file 1471-2148-11-334-S3.PDF]

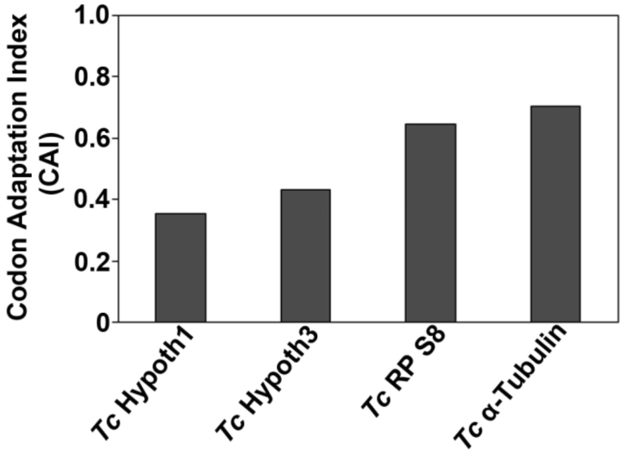

Figure S1: Codon Adaptation Index (CAI) of *META1* homologs in *T. cruzi*. Ribosomal Protein (RP) S8 and α-Tubulin have been used as reference genes. The accession numbers of the protein sequences used are: *Tc* Hypoth1: XP_814398.1; *Tc* Hypoth3: XP_802595.1; *Tc* RP S8: TcCLB.511903.110 and *Tc* α-Tubulin: TcCLB.411235.9.
