# Supplementary material for: Evolutionary and functional insights into Leishmania META1: evidence for lateral gene transfer and a role for META1 in secretion
Source: BMC Evol Biol. 2011 Nov 17;11:334. doi: 10.1186/1471-2148-11-334 (PMC3270026; doi:10.1186/1471-2148-11-334)
Supplement: Additional file 4 — Quantitation of signal in western blots in Figure 5. Figure S2. Images of western blots in Figure 5B, C and 5D were quantitated by ImageJ software. [file 1471-2148-11-334-S4.PDF]

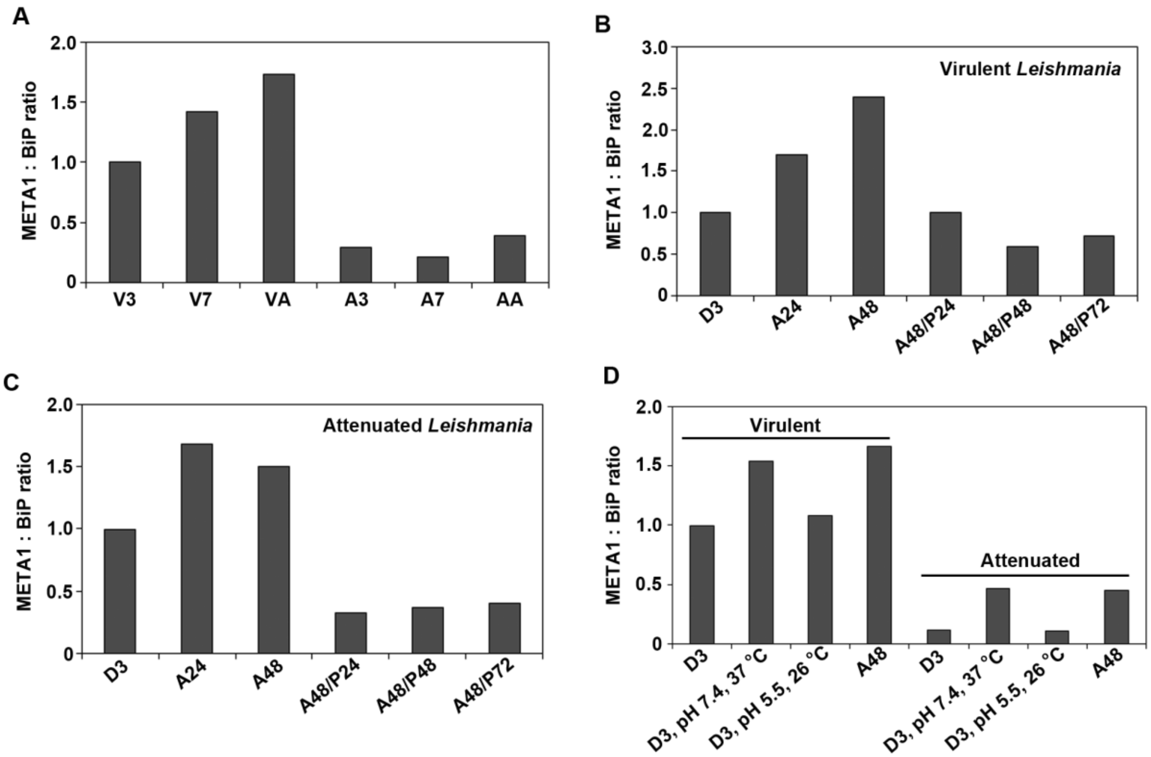

Figure S2: Quantitation of signal in western blots in Figure 5. Images of western blots in Figure 5B, C and D were quantitated by ImageJ software **[78]**. The values represented are amounts of META1 normalized to loading control BiP in each sample. **(A)** Quantitation for blot in Figure 5B. **(B)** and **(C)** Quantitation for blots in Figure 5C. **(D)** Quantitation for blot in Figure 5D.
