# Supplementary figures and images for: Evolutionary and functional insights into Leishmania META1: evidence for lateral gene transfer and a role for META1 in secretion
Source: BMC Evol Biol. 2011 Nov 17;11:334. doi: 10.1186/1471-2148-11-334 (PMC3270026; doi:10.1186/1471-2148-11-334)

## Slide 1
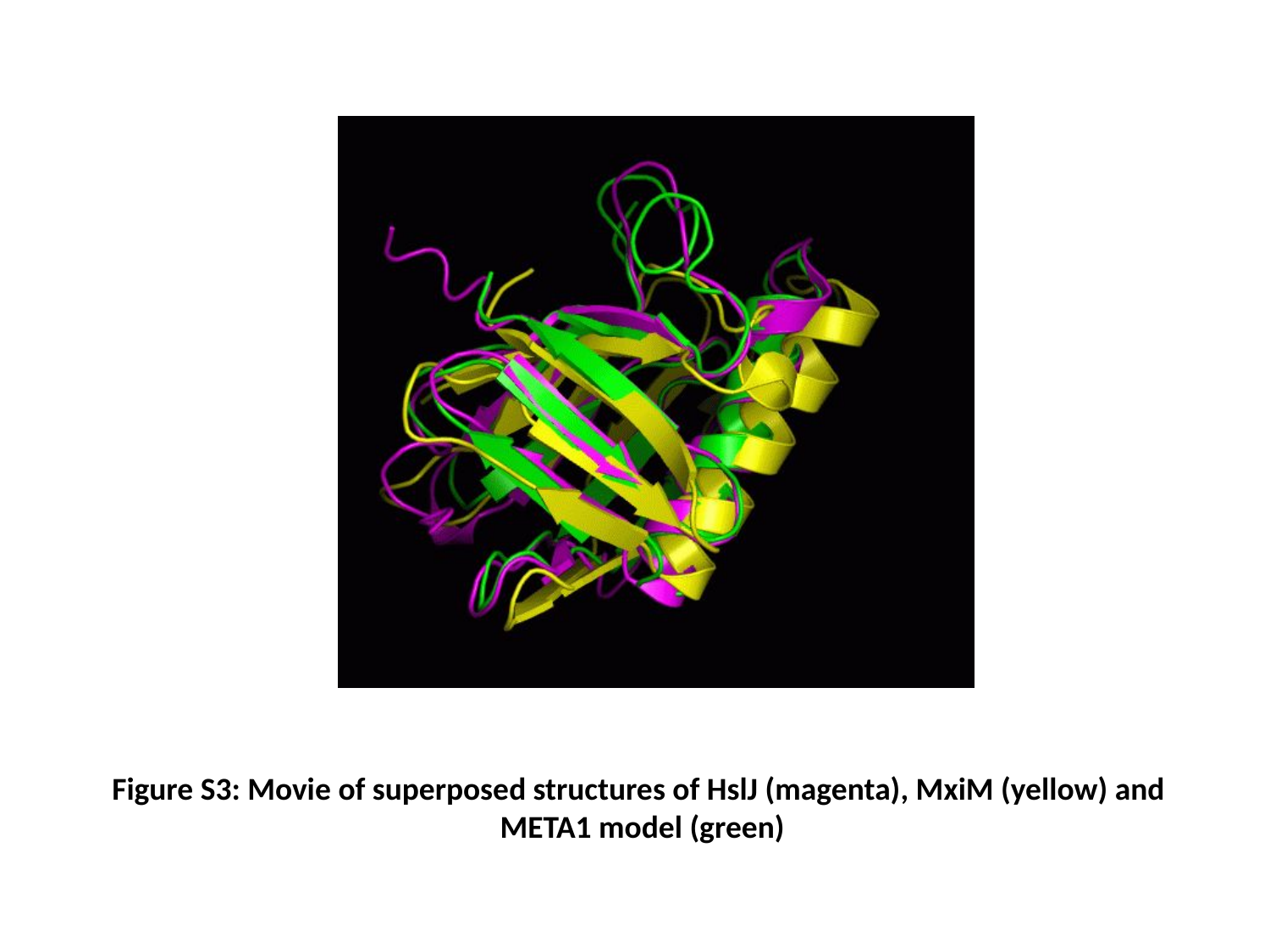

Figure S3: Movie of superposed structures of HslJ (magenta), MxiM (yellow) and
META1 model (green)

Supplement: Additional file 5 — Movie of superposed structures of HslJ (magenta), MxiM (yellow) and META1 model (green). Figure S3. [file 1471-2148-11-334-S5.PPT]
