# Supplementary material for: Evolutionary and functional insights into Leishmania META1: evidence for lateral gene transfer and a role for META1 in secretion
Source: BMC Evol Biol. 2011 Nov 17;11:334. doi: 10.1186/1471-2148-11-334 (PMC3270026; doi:10.1186/1471-2148-11-334)
Supplement: Additional file 6 — Overexpression of META1 in L. donovani. Figure S4. Western blots (S4A and S4B) of L. donovani virulent and attenuated META1 transfectants with META1, BiP and GFP antibodies. S4C represents effect of META1 overexpression on growth kinetics of virulent and attenuated L. donovani compared to their respective wild-types. [file 1471-2148-11-334-S6.PDF]

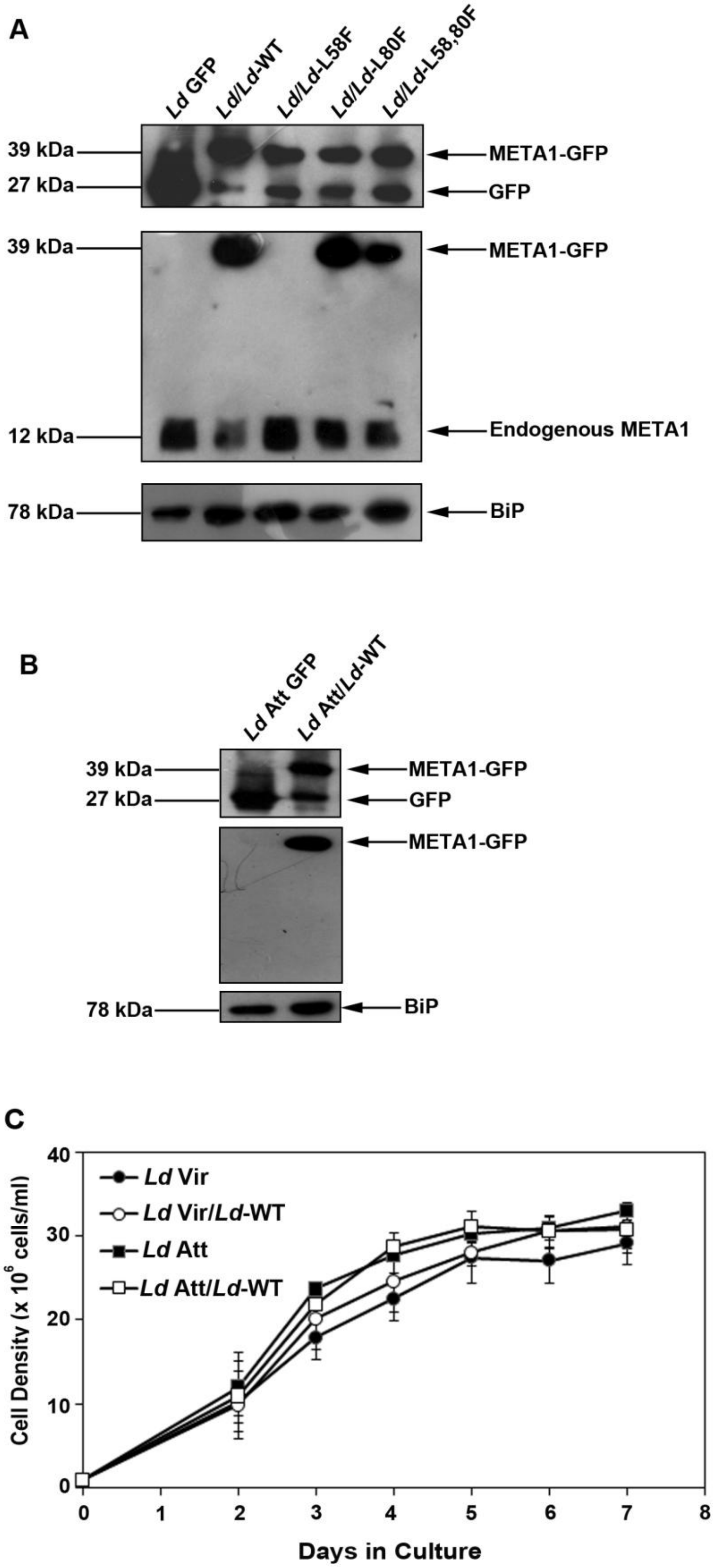
A
Ld GFP
Ld/Ld-WT
Ld/Ld-L58F
Ld/Ld-L80F
Ld/Ld-L58,80F
39 kDa
27 kDa
META1-GFP
GFP
39 kDa
META1-GFP
12 kDa
Endogenous META1
78 kDa
BiP
B
Ld Att GFP
Ld Att/Ld-WT
39 kDa
27 kDa
META1-GFP
GFP
META1-GFP
78 kDa
BiP
C
Ld Vir
Ld Vir/Ld-WT
Ld Att
Ld Att/Ld-WT
Cell Density (x 10^6 cells/ml)
Days in Culture
0
10
20
30
40
0
1
2
3
4
5
6
7
8

Figure S4: Overexpression of META1 in *L. donovani*. For western blots, **t**otal cell lysates from stationary phase of *Leishmania* were obtained. The samples were assessed by western blotting with anti-GFP (upper panel), anti-META1 (middle panel) and anti-BiP (lower panel) antibodies. *Leishmania* BiP is a chaperone protein in the endoplasmic reticulum and was used as a loading control. Ectopically expressed META1 was tagged with GFP at the C-terminus. **(A)** Virulent *L. donovani* META1-GFP overexpression lines (wild-type & mutant) compared to vector control, *Ld* GFP. Lane 1: *Ld* GFP; Lane 2: *Ld/Ld*-WT; Lane 3: *Ld/Ld*-L58F; Lane 4: *Ld/Ld*-L80F and Lane 5: *Ld/Ld*-L58,80F. **(B)** Attenuated *L. donovani* META1-GFP overexpression line compared to vector control, *Ld* Att GFP. Lane 1: *Ld* Att GFP and Lane 2: *Ld* Att/*Ld*-WT. **(C)** Effect on growth kinetics in *L. donovani* on META1 overexpression. Growth curve of virulent and attenuated *L. donovani* overexpressing wild-type META1, *Ld* Vir*/Ld*-WT and *Ld* Att*/Ld*-WT respectively were compared to their respective control lines, *Ld* Vir and *Ld* Att. Cell density (x 10^6 cells/ml) of each culture was determined at 24 hour time intervals after 48 hours of initial inoculation for up to 7 days. The cell densities plotted are average of at least 3 biological replicates.
