# Supplementary material for: Evolutionary and functional insights into Leishmania META1: evidence for lateral gene transfer and a role for META1 in secretion
Source: BMC Evol Biol. 2011 Nov 17;11:334. doi: 10.1186/1471-2148-11-334 (PMC3270026; doi:10.1186/1471-2148-11-334)
Supplement: Additional file 7 — Mutagenesis of META1. Figure S5. Sequence alignment of META1 homologs and stereoscopic representation of META1 model highlighting the leucine 58 and leucine 80. [file 1471-2148-11-334-S7.PDF]

A

```
MxiM        -------------------S--SSNSEKEWHIVPVSKDYFS--IPNDLLWSFNTTNKSINVYSKCIS-GK 46
HslJ        ------------------MVTPEQLQHHRFVLESVNGKPVT-SDKNPPEISFGEKM---MISGSMCNRFS 48
Tbhypoth1   MKETLLLFLPTFKPTRITMATCPKHLFGKYHLKSFNGKSALEGELAGVTLELGEEDGRVTVRAKAGNAMS 70
LbrMETA1    ------------------MG--IEDLHGKYTAKFMDGVPAP--AGVTVEFSAGANSGAVRIHARVVNIMN 48
LinfMETA1   ------------------ME--MKNLLGKHKVVLVNGRPAP--AGVTVEFKAGENSGSVHMHARVANIMN 48
LmexMETA1   ------------------ME--MKSLIGKYRVLSVNGRPAP--AGVTVEFKPGENSGTIQMHAKVANFMN 48
LamMETA1    ------------------ME--MKSLIGKRRVLSVNGRPAP--AGVTVEFKASENSGTIQMHANVANFMN 48
LmjMETA1    ------------------ME--MKNLLGKHKIVSVNGKPAP--AGVTVEFKASENSGSVYMHAKVANIMN 48
LdMETA1     ------------------ME--MKNLLGKHRVVSVNGRPAP--AGVTVEFKASENSGFVHMHARVANIMN 48
Tchypoth1   ------------------MSSIPDELFGNWLLTTFNGLDLS-DQGGPFLMLLEPAGGLVYLDIQITNILN 51

MxiM        AVYSFNAGKFM-GNFNVKEVDGCFMDAQKIAIDKLF-SMLKDG--VVLKGNKINDTILIEKDGEVKLKLI 112
HslJ        GEGKLSNGELTAKGL-AMTRMMC-ANPQLNELDNTISEMLKEGAQVDLTAN----QLTLATA-KQTLTYK 111
Tbhypoth1   GVLKYEGGMLT-GPL-MSTMMMP--PPAVLKVERTLVSGFSSGMHALREGS----SLTLSHK-TDTMVFE 131
LbrMETA1    GVLKVKENKLS-GSL-ISTMMAG--SDDLMAIEGIFLQGFTSGVTYTLREDG---KLVLQLK-SHTIEFV 110
LinfMETA1   GQLRLVNRKLS-GAL-VSTMMLG--SDDLMNIENALSQGFMEGMTYTVKDGG---KLTLQSK-THTIKLV 110
LmexMETA1   GQLKLGNRKLS-GTL-VSTMMLG--SDDLMNIENALSQGFMDGMAYTVHDGG---KLTLKSN-THTIKLV 110
LamMETA1    GQLKLGNRKLS-GTL-VSTMMLG--SDDLMNIENALIQGFMDGMTYTVHDGG---KLTLKSN-THTIKLV 110
LmjMETA1    GPLKLANRKLS-GAL-VSTMMLG--SDDLMNIENALSQGFMEGMTYTVKDGG---KLTLQSK-THIIMLV 110
LdMETA1     GPLRLENRKLS-GAL-VSTMMLG--SDDLMNIENALSQGFIEGMTYAVKDGG---KLTLQSK-THTIKLV 110
Tchypoth1   GLLEYRDGQLY-GHL-VSTRMLG--PPLHMQIEQAFGAGFEAGMNVLIEEA----GMIFSQG-GNTFFFV 112

MxiM        RGI----- 115
HslJ        LADLMN-- 117
Tbhypoth1   AVE----- 134
LbrMETA1    PA------ 112
LinfMETA1   PA------ 112
LmexMETA1   PA------ 112
LamMETA1    PA------ 112
LmjMETA1    PA------ 112
LdMETA1     PA------ 112
Tchypoth1   AQTDSSQQ 120
```

B

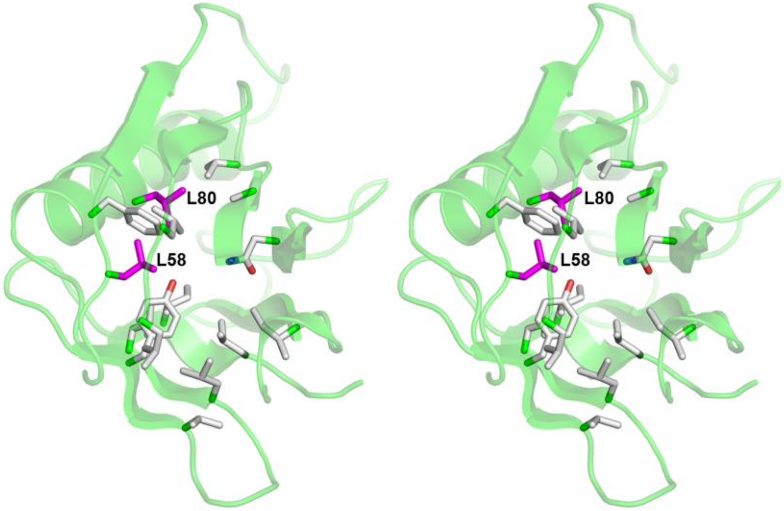

Figure S5: Mutagenesis of META1. (A) Sequence to structure alignment of MxiM (1y9t), HslJ (2kts) and META1 homologs in trypanosomatids, with leucine 58 & leucine 80 highlighted that were mutated. The numbers on the right indicate amino acid positions. The alignment was generated by PROMALS3D **[63]**. **(B)** Stereoscopic representation of META1 model showing the side chains of the residues lining the hydrophobic cavity. Carbon atoms are coloured in gray, nitrogen atoms are colored in blue and oxygen atoms are coloured in red. Leucine 58 & leucine 80 have been highlighted in magenta. The figure was prepared using the software PyMol **[73]**.
