# Supplementary material for: Evolutionary and functional insights into Leishmania META1: evidence for lateral gene transfer and a role for META1 in secretion
Source: BMC Evol Biol. 2011 Nov 17;11:334. doi: 10.1186/1471-2148-11-334 (PMC3270026; doi:10.1186/1471-2148-11-334)
Supplement: Additional file 8 — Effect of META1 overexpression in L. major. Figure S6. Effect of WT and mutant META1 overexpression in L. major on extracellular SAP activity (S6A) and growth kinetics (S6B). S6C and S6D represents western blot and QRT-PCR of L. major META1 transfectants respectively. [file 1471-2148-11-334-S8.PDF]

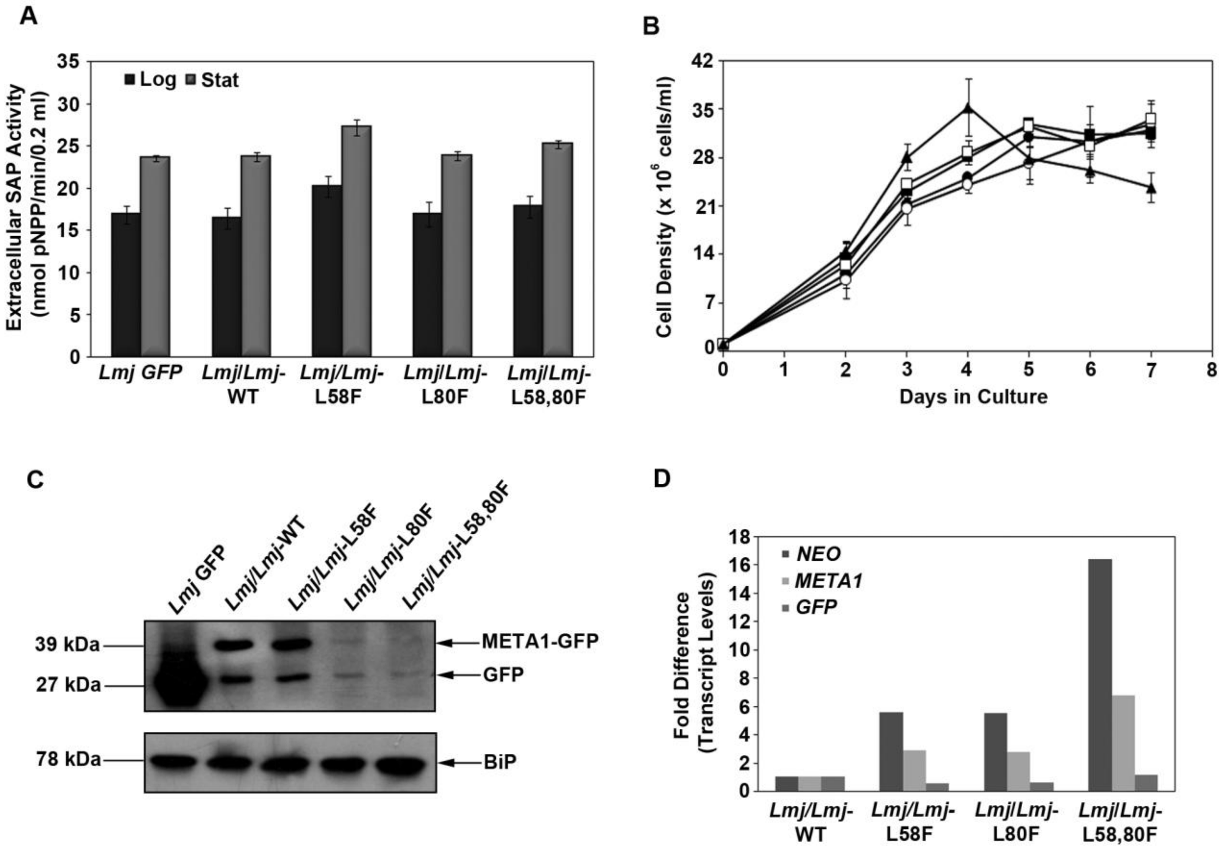

Figure S6: Effect of META1 overexpression in *L. major.* **(A)** Effect on extracellular SAP activity on META1 overexpression. SAP activity in *L. major* at log (black bars) and stationary phase (grey bars) of vector control (*Lmj* GFP), wild-type META1 overexpression line (*Lmj/Lmj*-WT), mutant META1 overexpression lines: L58F (*Lmj/Lmj*-L58F); L80F (*Lmj/Lmj*-L80F) and L58,50F (*Lmj/Lmj*-L58,80F) respectively. SAP activity is represented in nmoles of pNPP hydrolyzed per min per 0.2 ml. The average of at least 3 biological replicates is presented. Bar represents the standard deviation. **(B)** Effect on growth kinetics in *L. major* on META1 overexpression. Growth curves of *L. major* overexpressing either the wild-type META1, *Lmj/Lmj*-WT (white circle) or mutant META1 L58F (black square), L80F (white square) and L58,80F (black triangle) were compared to that of *Leishmania* with control vector, *Lmj* GFP (black circle). Cell density (x 10^6 cells/ml) of each culture was determined at 24 hour time intervals after 48 hours of initial inoculation for up to 7 days. The cell densities plotted are average of at least 3 biological replicates. **(C)** Western blot of *L. major* META1 overexpression lines. *L. major* META1-GFP overexpression (wild-type & mutant) compared to vector control, *Lmj* GFP. Lane 1: *Lmj* GFP; Lane 2: *Lmj/Lmj*-WT; Lane 3: *Lmj/Lmj*-L58F; Lane 4: *Lmj/Lmj*-L80F and Lane 5: *Lmj/Lmj*-L58,80F. Upper and lower panels were probed with anti-GFP and anti-BiP respectively. **(D)** Transcript levels of *META1, GFP* and *NEO* (neomycin). QRT-PCR analysis of META1 overexpression lines (wild-type & mutant) for transcript levels of *META1*, *GFP* and *NEO*. The relative expression of the 3 genes is presented as fold difference over expression in *Lmj/Lmj*-WT.
