# Supplementary material for: Evolutionary and functional insights into Leishmania META1: evidence for lateral gene transfer and a role for META1 in secretion
Source: BMC Evol Biol. 2011 Nov 17;11:334. doi: 10.1186/1471-2148-11-334 (PMC3270026; doi:10.1186/1471-2148-11-334)
Supplement: Additional file 9 — Western blot of L. donovani whole cell lysates v/s culture supernatants. Figure S7. Western blot with GP63 and BiP on whole cell lysates and culture supernatants of L. donovani META1 transfectants. [file 1471-2148-11-334-S9.PDF]

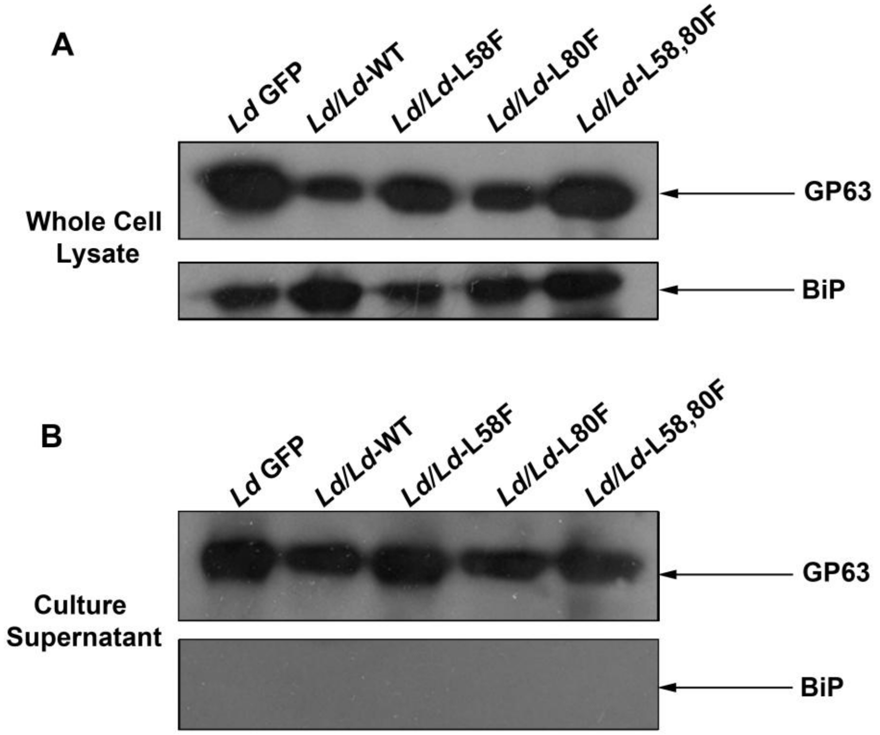

Figure S7: Western blot of *L. donovani* whole cell lysates v/s culture supernatants. (A) Whole cell lysates of *L. donovani* transfectants and **(B)** Concentrated culture supernatants of *L. donovani* transfectants were used for western blot against GP63 (upper panels in A & B) and BiP (lower panels in A & B) antibodies.
