# Supplementary material for: Evolutionary and functional insights into Leishmania META1: evidence for lateral gene transfer and a role for META1 in secretion
Source: BMC Evol Biol. 2011 Nov 17;11:334. doi: 10.1186/1471-2148-11-334 (PMC3270026; doi:10.1186/1471-2148-11-334)
Supplement: Additional file 10 — Phylogenetic tree re-rooted at two different roots. Figure S8. Phylogenetic tree representation generated in Figure 3 was re-rooted at Tolumonas (S8A) and T. cruzi Hypoth3 (S8B). [file 1471-2148-11-334-S10.PDF]

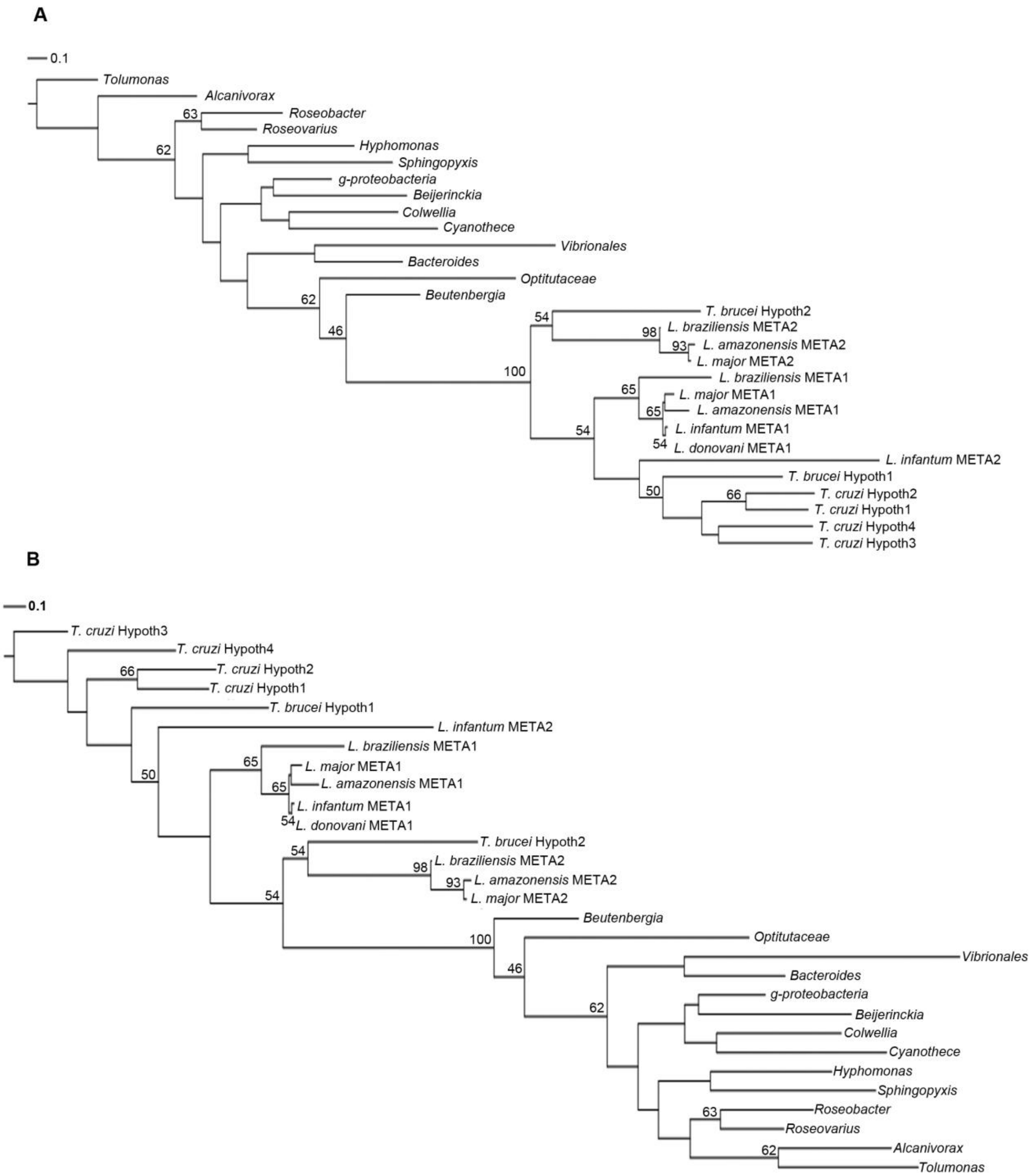

Figure S8: Phylogenetic tree re-rooted at two different roots. Phylogenetic tree representation generated in Figure 3 was re-rooted at **(A)** *Tolumonas* and **(B)** *T. cruzi* Hypoth3 using iTOL **[70]**. A horizontal bar with 0.1 above each tree represents scale of the generated tree.
