# Supplementary material for: Evolutionary and functional insights into Leishmania META1: evidence for lateral gene transfer and a role for META1 in secretion
Source: BMC Evol Biol. 2011 Nov 17;11:334. doi: 10.1186/1471-2148-11-334 (PMC3270026; doi:10.1186/1471-2148-11-334)
Supplement: Additional file 11 — List of protein sequences used in phylogenetic analysis. Table S3. Accession numbers of protein sequences. [file 1471-2148-11-334-S11.PDF]

Table S3. List of protein sequences used in phylogenetic analysis in Figure 3

Gene Name	Accession No.
L. major META1	XP_001682347.1
L. donovani META1	AAC04778.1
L. infantum META1	XP_001464758.1
L. amazonensis META1	AAC04758.1
L. braziliensis META1	XP_001563865.1
L. major META2	XP_001682345.1
L. infantum META2	XP_001464756.1
L. amazonensis META2	AAL32493.2
L. braziliensis META2	XP_001563874.1
T. cruzi Hypoth1	XP_814398.1
T. cruzi Hypoth2	XP_802595.1
T. cruzi Hypoth3	XP_814407.1
T. cruzi Hypoth4	XP_808205.1
T. cruzi Hypoth5	XP_814405.1
T. brucei Hypoth1	XP_844890.1
T. brucei Hypoth2	XP_844893.1
Cyanothece	ZP_03153678.1
Beutenbergia	YP_002881157.1
Tolumonas	YP_002892522.1
gamma-proteobacterium	ZP_05061049.1
Roseobacter	ZP_01901204.1
Sphingopyxis	YP_617096.1
Alcanivorax	ZP_05041611.1
Vibrionales	XP_844891.1
Roseovarius	XP_844900.1
Colwellia	XP_001464756.1
Beijerinckia	YP_001832384.1
Bacteroides	YP_001299662.1
Hyphomonas	YP_759703.1
Opitutaceae	ZP_03723590.1
