# Supplementary material for: Evolutionary and functional insights into Leishmania META1: evidence for lateral gene transfer and a role for META1 in secretion
Source: BMC Evol Biol. 2011 Nov 17;11:334. doi: 10.1186/1471-2148-11-334 (PMC3270026; doi:10.1186/1471-2148-11-334)
Supplement: Additional file 12 — Primers used in the study. Table S4. List of sequences of all the primers used in this study. [file 1471-2148-11-334-S12.PDF]

Table S4. Primers used in the study

Primer	Sequence	Remarks
TV359	5'-TAGATACACGCGCCCTGTCTTATT-3'	*LdMETA1*-F for RT-PCR
TV360	5'-CTGTTGATGATGGCCATGCGCGT-3'	*LdMETA1*-R for RT-PCR
TV672	5'- CATGAACGGCCCGTTGAGGCT-3'	*LmjMETA1*-F for RT-PCR
TV397	5'-CTCCTGCCGAGAAAGTATCCA-3'	*NEO*-F for RT-PCR
TV398	5'-ACAGTTCGGCTGGCGCGAG-3'	*NEO*-R for RT-PCR
TV396	5'-GTGGTCACGAGGGTGGGCCA-3'	*GFP*-R for RT-PCR
TV366	5'-AGTACACGGTGGAGGCTGTG-3'	*GAPDH*-F for RT-PCR
TV367	5'-ACCCTTGATGTGACCCTCGG-3'	*GAPDH*-R for RT-PCR
TV213	5'-CCGGAATTCATGAAACACTTGCTTGGCAA-3'	*LdMETA1*-F for pET28a+ cloning
TV215	5'-CCCAAGCTTGCATCTACGCAGGAACGA-3'	*LdMETA1*-R for pET28a+ cloning
TV276	5'-GCCACCATGGTGAGCAAGGGCGAG-3'	*GFP*-F for cloning and RT-PCR
TV277	5'-GCTCTAGATTACTTGTACAGCTGC-3'	*GFP*-R for cloning
TV494	5'-ATGCAGGATCCTATGGAGATGAAAAACTTG-3'	*META1*-F for cloning
TV757	5'-CCATGGACGCAGGAACAAGCTTGATG-3'	*LdMETA1*-R for cloning
TV758	5'-CCATGGACGCAGGAACAAGCATAATGATAT-3'	*LmjMETA1*-R for cloning
TV868	5'-CTCGAAAACCGCAAGTTCTCTGGTGCTCTCG-3'	*Ld*-L58F-F for SDM
TV869	5'-CGAGAGCACCAGAGAACTTGCGGTTTTCGAG-3'	*Ld*-L58F-R for SDM
TV870	5'-CTGATGAATATCGAGAACGCATTCAGTCAGGGATTCATTG-3'	*Ld*-L80F-F for SDM
TV871	5'-CAATGAATCCCTGACTGAATGCGTTCTCGATATTCATCAG-3'	*Ld*-L80F-R for

		SDM
TV872	5’-CTCGCAAACCGCAAGTTCTCTGGTGCTCTTG-3’	*Lmj*-L58F-F for SDM
TV873	5’-CAAGAGCACCAGAGAACTTGCGGTTTGCGAG-3’	*Lmj*-L58F-R for SDM
TV874	5’-TGAATATCGAGAACGCCTTCAGTCAGGGATTCATG-3’	*Lmj*-L80F-F for SDM
TV875	5’-CATGAATCCCTGACTGAAGGCGTTCTCGATATTCA-3’	*Lmj*-L80F-R for SDM
